# Monitoring Solution Structures of Peroxisome Proliferator-Activated Receptor β/δ upon Ligand Binding

**DOI:** 10.1371/journal.pone.0151412

**Published:** 2016-03-18

**Authors:** Rico Schwarz, Dirk Tänzler, Christian H. Ihling, Andrea Sinz

**Affiliations:** Department of Pharmaceutical Chemistry and Bioanalytics, Institute of Pharmacy, Martin Luther University Halle-Wittenberg, D-06120, Halle/Saale, Germany; Philipps University, GERMANY

## Abstract

Peroxisome proliferator-activated receptors (PPARs) have been intensively studied as drug targets to treat type 2 diabetes, lipid disorders, and metabolic syndrome. This study is part of our ongoing efforts to map conformational changes in PPARs in solution by a combination of chemical cross-linking and mass spectrometry (MS). To our best knowledge, we performed the first studies addressing solution structures of full-length PPAR-β/δ. We monitored the conformations of the ligand-binding domain (LBD) as well as full-length PPAR-β/δ upon binding of two agonists. (Photo-) cross-linking relied on (i) a variety of externally introduced amine- and carboxyl-reactive linkers and (ii) the incorporation of the photo-reactive amino acid *p*-benzoylphenylalanine (Bpa) into PPAR-β/δ by genetic engineering. The distances derived from cross-linking experiments allowed us to monitor conformational changes in PPAR-β/δ upon ligand binding. The cross-linking/MS approach proved highly advantageous to study nuclear receptors, such as PPARs, and revealed the interplay between DBD (DNA-binding domain) and LDB in PPAR-β/δ. Our results indicate the stabilization of a specific conformation through ligand binding in PPAR-β/δ LBD as well as full-length PPAR-β/δ. Moreover, our results suggest a close distance between the *N*- and *C*-terminal regions of full-length PPAR-β/δ in the presence of GW1516. Chemical cross-linking/MS allowed us gaining detailed insights into conformational changes that are induced in PPARs when activating ligands are present. Thus, cross-linking/MS should be added to the arsenal of structural methods available for studying nuclear receptors.

## Introduction

(Photo-) chemical cross-linking combined with mass spectrometry (MS) has evolved as an alternative method to obtain low-resolution three-dimensional (3D) structural information of proteins and protein complexes.[1–9] The cross-linking/MS approach allows deriving structural information by covalently connecting pairs of functional groups in the protein(s) under investigation. This work follows up on a previous study, in which the distances bridged by different cross-linkers as well as an incorporated photo-reactive amino acid had served as “molecular rulers” to map the 3D-structures of free and ligand-bound peroxisome proliferator-activated receptor alpha (PPAR-α). Using chemical cross-linking/MS, we had been able to monitor conformational changes in the ligand-binding domain (LBD) of PPAR-α upon ligand binding.[8]

**Fig 1:**
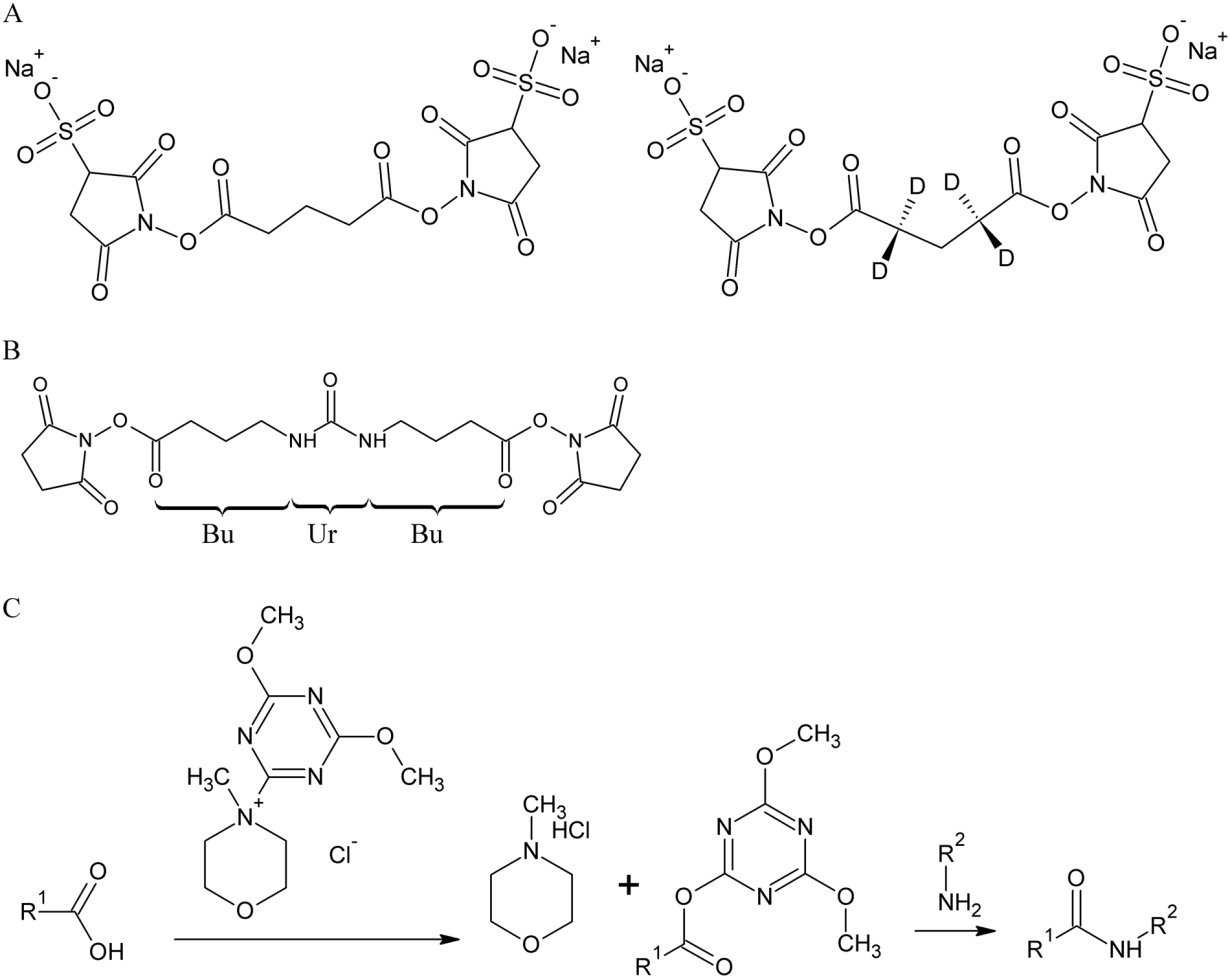
Structures and reaction mechanisms of the cross-linkers used in this study. (A) Amine-reactive, isotope-labeled cross-linker BS^2^G-*D*_*0*_/*D*_*4*_. (B) MS/MS-cleavable amine-reactive urea-linker; cleavable bonds are indicated in red; the fragments created under MS/MS conditions are denoted as “Bu” and “BuUr” according to [15]. (C) Reaction mechanism of the zero-length cross-linker DMTMM, connecting carboxyl and amine groups in proteins.

In the present work, we aim to monitor conformational changes in the PPAR-β/δ isoform. For this, we applied the amine-reactive cross-linker *bis*(sulfosuccinimidyl)glutarate (BS^2^G; Fig 1A) that bridges Cα-Cα distances up to 27 Å.[10, 11] BS^2^G is an *N*-hydroxysuccinimide (NHS) ester that connects lysines—as well as the *N*-terminus—in a protein, but it also possesses a tendency to react with serines, threonines, and tyrosines.[12] As an additional amine-reactive cross-linker we used the *in-house* synthesized urea-cross-linker (Fig 1B).[13] This linker possesses unique properties for an automated identification of cross-linked products based on its characteristic fragmentation patterns that are created upon collision-induced dissociation (CID)-tandem MS conditions. Also, cross-linking experiments were performed with the zero-length cross-linker DMTMM (4-(4,6-dimethoxy-1,3,5-triazin-2-yl)-4-methylmorpholinium chloride; Fig 1C) that is able to connect amines (*N*-terminus and lysines) with carboxyl groups (glutamic acid and aspartic acid). In a recent study, DMTMM was employed as activating reagent to couple carboxyl groups in proteins by a hydrazide cross-linker.[14]

**Fig 2:**
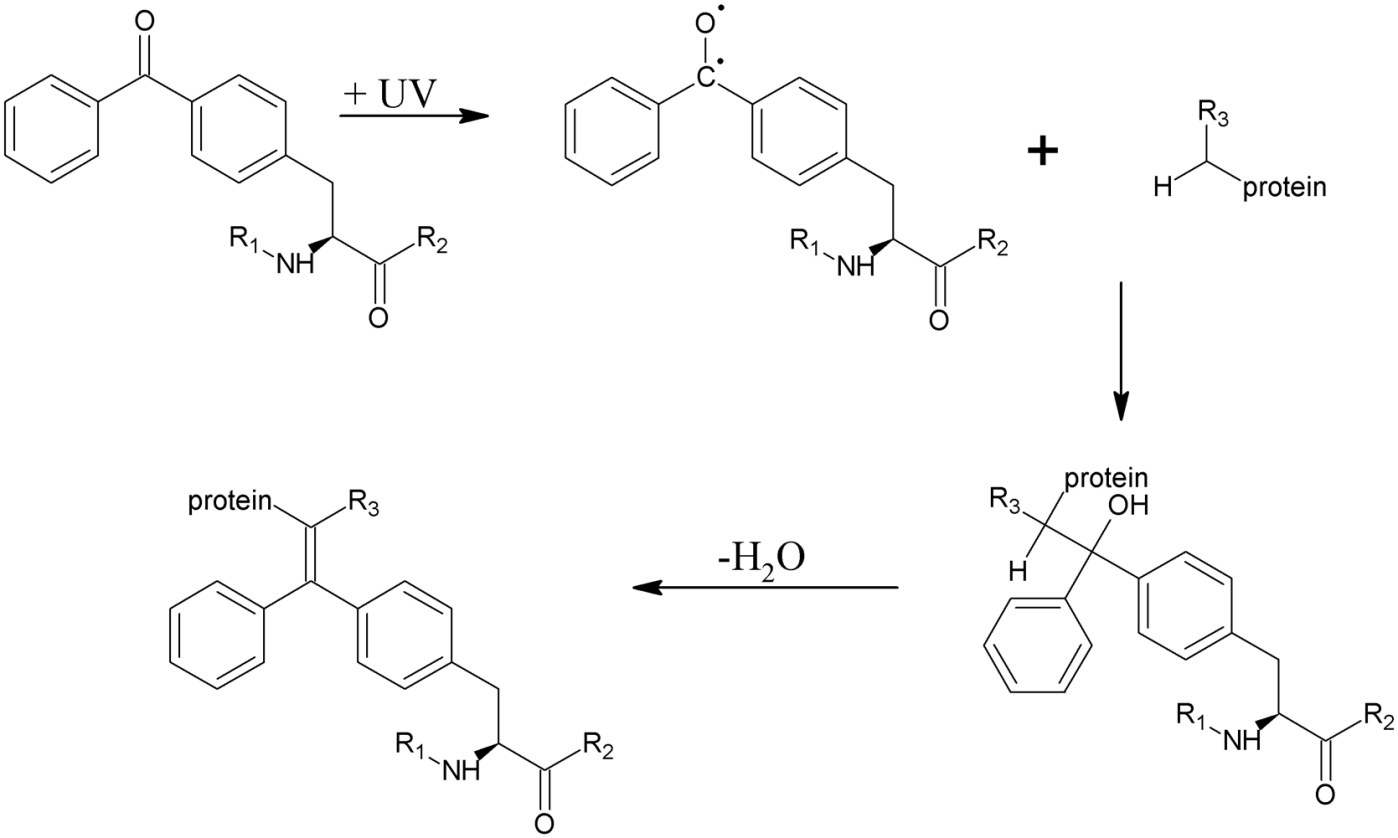
Reaction scheme of the photo-reactive amino acid Bpa. After photo-cross-linking, the reaction product might lose a water molecule during MS analysis.

In addition, we performed photo-cross-linking experiments by incorporating the unnatural photo-activatable amino acid *para*-benzoyl-L-phenylalanine (Bpa) at specific positions into the LBD of PPAR-β/δ.[16] The benzophenone group of Bpa is activated by UV-A light and can potentially insert into CH, NH, SH and OH groups of all 20 proteinogenic amino acids (Fig 2); yet, it has been shown that Bpa possesses a certain preference towards methionines.[17, 18]

PPARs are ligand-activated transcription factors that belong to the nuclear receptor protein family. So far, three subtypes of PPARs (α, β/δ, γ) have been identified.[19–21] They are composed of a DNA-binding domain (DBD) and a ligand-binding domain (LBD; for amino acid sequence, please see S1 Fig). PPARs are activated by fatty acids and eicosanoids, but also by a number of low-molecular weight compounds.[22–26] After activation, PPARs form heterodimers with the retinoid X receptor and bind to specific DNA sequences.[27, 28] In general, PPAR-α promotes fatty acid catabolism in the liver and the skeletal muscle, while PPAR-γ regulates fatty acid storage in adipose tissues.[29–31] PPAR-β/δ is expressed ubiquitously [32] and is involved in fatty acid catabolism,[33] cell differentiation,[34] and cancer.[35] Due to its complex roles in the human metabolism, PPAR-β/δ is an attractive target for drug design.

**Fig 3:**
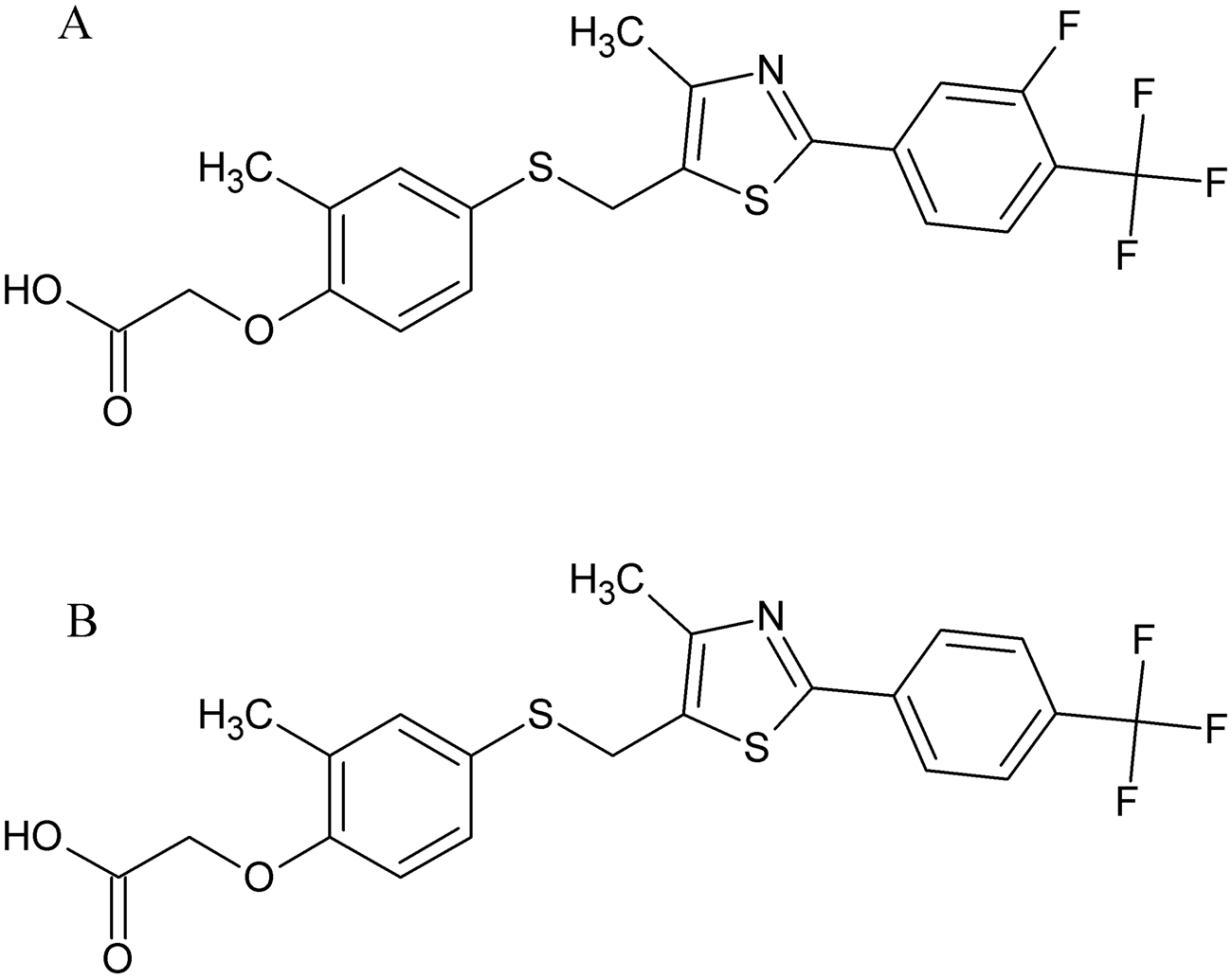
Structures of PPAR-β/δ agonists (A) GW0742 and (B) GW1516.

**Fig 4:**
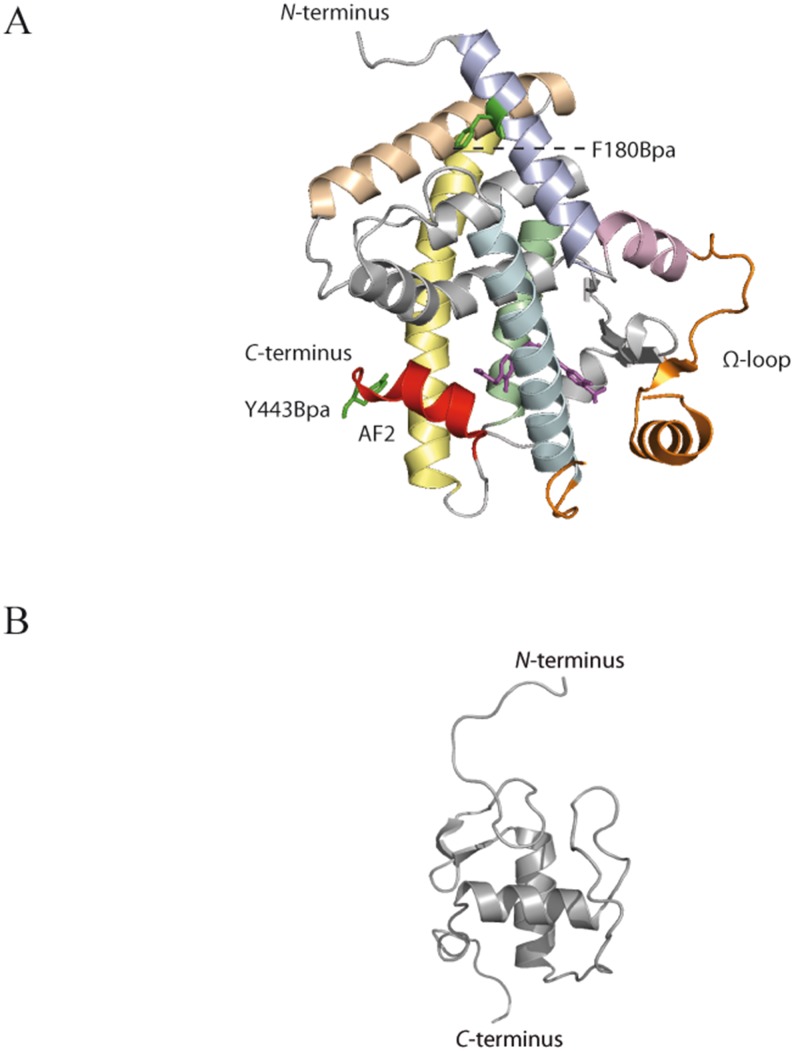
High-resolution 3D-structures of PPAR-β/δ. (A) X-ray structure of PPAR-β/δ LBD (pdb 3TKM), bound to agonist GW0742; The activation function helix 2 (AF2, helix 12) is shown in red, the flexible Ω-loop is shown in orange; the amino acids replaced by Bpa are shown as green sticks; the ligand GW0742 is shown in stick representation in magenta. Helices containing amino acids that are involved in cross-linking are colored (helix 1: light blue; helix 2: light pink; helix 4: pale cyan; helix 8: pale green; helix 10: wheat; helix 11: pale yellow). (B) NMR structure of PPAR-β/δ DBD (pdb 2ENV).

Here, we study the conformational changes in the LBD as well as full-length PPAR-β/δ upon binding of the agonists GW0742 (K_D_ = 0.4 nM, EC_50_ = 1 nM) and GW1516 (K_D_ = 1.1 nM, EC_50_ = 1 nM) (Fig 3).[22] After (photo-) chemical cross-linking, enzymatic digestion, and MS analysis of the cross-linked peptides, the distance constraints obtained allowed us to study the conformational changes upon ligand binding in PPAR-β/δ. The identified cross-links in the DBD and the LBD were in good agreement with published NMR and X-ray 3D-structures (pdb entries 2ENV and 3TKM; Fig 4).[36] With the (photo-) cross-linking/MS approach, we obtained detailed structural information mainly from the flexible *N*-terminal region and the hinge region of full-length PPAR-β/δ upon ligand binding.

## Material and Methods

### Reagents and Chemicals

Agonists GW0742 and GW1516 were purchased from Sigma-Aldrich (Taufkirchen, Germany). Nano-HPLC solvents were LC-MS grade (VWR, Darmstadt, Germany), water was purified with a TKA X-CAD system (Thermo Fisher Scientific, Bremen, Germany). Trypsin (cleaving *C*-terminally of lysine and arginine), GluC (cleaving *C*-terminally of glutamate and aspartate), and ProTEV Plus (cleaving *C*-terminally of ENLYFQ(G/S)) were obtained from Promega (Mannheim, Germany). The cross-linker BS^2^G-*D*_*0*_/*D4* was obtained from Thermo Fisher Scientific (Rockford, IL). The urea-linker was synthesized and purified *in-house*.[13] Bpa was obtained from Bachem (Bubendorf, Switzerland). Tryptone, yeast extract, antibiotics, TCEP, and IPTG were purchased from Roth (Karlsruhe, Germany). Iodacetamide, DTT, D-desthiobiotin, DMTMM, and all other chemicals (Sigma-Aldrich, Taufkirchen, Germany) were obtained at the highest available purity.

### Protein Expression and Purification

Expression of PPAR-β/δ LBD as *N*-terminal Strep-tag II construct was performed as described recently.^8^ The photo-activatable amino acid Bpa was incorporated into PPAR-β/δ at positions Phe-180 and Tyr-443 according to the method described by Schultz *et al*. [16] Cells were resuspended in buffer A (50 mM HEPES, 300 mM NaCl, 1 mM TCEP, pH 6.8) at a 1:3 ratio. To resuspended cells, one tablet of protease inhibitor complete (Roche, Mannheim, Germany) and benzonase (final concentration 5 U/ml; Sigma-Aldrich, Taufkirchen, Germany) were added. After sonication on ice, the lysate was centrifuged at 35,000 g (4°C, 60 min). Before chromatographic separation, the supernatant was filtered using Filtropur S 0.2 μm filtration units (Sarstedt, Nümbrecht, Germany). The purification step was carried out on an ÄKTA Prime system (GE Healthcare, Munich, Germany) at 6°C. The supernatant was loaded at 0.3 ml/min onto two connected 1 ml StrepTrap HP columns (GE Healthcare, Munich, Germany) using a 50-ml superloop. Wildtype PPAR-β/δ and variants and were eluted with 1 ml/min buffer B (50 mM HEPES, 300 mM NaCl, 1 mM TCEP, 10 mM D-desthiobiotin, pH 6.8). PPAR-β/δ-containing fractions were pooled, washed, and concentrated via Amicon ultracentrifugation units (cut-off 10 kDa; Millipore, Darmstadt, Germany) to 12−35 μM and stored at −20°C. Full-length PPAR-β/δ was obtained from Biomol (Hamburg, Germany) as *N*-terminal (His)_6_ construct (for amino acid sequence please see S1 Fig).

### Cross-Linking Experiments

TEV protease was added (5–10 U) to PPAR-β/δ LBD wildtype and variants as well as full-length PPAR-β/δ to remove the tag (Strep-tag II for PPAR-β/δ LBD; (His)_6_-tag for full-length PPAR-β/δ). After cleavage (6°C, overnight) all samples were washed three times with buffer A (see Protein Expression and Purification) using an Amicon centrifugation unit (10 kDa). Samples were adjusted to a protein concentration of 5 μM and immediately used for (photo-) cross-linking experiments. For photo-cross-linking, the PPAR-β/δ LBD variants F180Bpa and Y443Bpa were mixed with the agonist GW0742 or GW1516 in DMSO to give a 20-fold molar excess of ligand over protein. As a control, one sample was mixed with DMSO without the addition of ligand. After 30 min at 4°C, the solutions were irradiated with UV-A light (365 nm; 8 J/cm^2^) to activate the photo-reactive amino acid Bpa (Scheme 1). Afterwards, samples were analyzed by SDS−PAGE or subjected to *in-solution* digestion with trypsin or a trypsin/GluC mixture. Bands of interest were excised from SDS-gels and *in-gel* digested with trypsin according to an existing protocol.[37] After enzymatic digestion, the peptide mixtures were immediately analyzed by LC/MS/MS.

PPAR-β/δ LBD and full-length PPAR-β/δ were mixed with both agonists as described above. After incubation on ice, the PPAR-β/δ LBD was mixed either with DMTMM (8000-fold molar excess over protein), BS^2^G-*D*_*0*_/*D*_*4*_ (100- or 200-fold molar excess over protein) or the urea-linker (100- or 200-fold molar excess over protein) for 2 hrs on ice. Samples were either quenched with ammonium bicarbonate (BS^2^G-*D*_*0*_/*D*_*4*_ and urea-linker) to a final concentration of 20 mM or immediately mixed with Laemmli buffer and analyzed by SDS-PAGE (DMTMM). Full-length PPAR-β/δ was mixed with BS^2^G-*D*_*0*_/*D*_*4*_ to give a 200-fold molar excess of cross-linker over the protein. After 2 hrs on ice, samples were quenched as described above.

### Nano-HPLC/Nano-ESI-Orbitrap-MS/MS Analysis

*In-solution* and *in-gel* digestion mixtures were analyzed by LC/MS on an UltiMate 3000 RSLC Nano system (Thermo Fisher Scientific, Bremen, Germany) coupled to an Orbitrap Fusion Tribrid mass spectrometer (Thermo Fisher Scientific, Bremen, Germany) equipped with a Nanospray Flex Ion Source (Thermo Fisher Scientific, Bremen, Germany). Samples were loaded onto a pre-column (C8 reversed phase, Acclaim PepMap, 300 μm * 5 mm, 5 μm, 100 Å, Thermo Fisher Scientific, Bremen, Germany) and washed with water containing 0.1% (v/v) TFA for 15 min, before the peptides were separated on the separation column (C18 reversed phase, Acclaim PepMap, 75 μm * 250 mm, 2 μm, 100 Å, Thermo Fisher Scientific, Bremen, Germany) using gradients from 1% to 35% (v/v) B (90 min), 35% to 85% (v/v) B (5 min) followed by 85% B (5 min), with solvent A: 0.1% (v/v) formic acid (FA) in water (LC-MS grade, VWR, Darmstadt, Germany) and solvent B: 0.08% (v/v) FA in acetonitrile (LC-MS grade, VWR, Darmstadt, Germany). Data were acquired using data-dependent MS/MS mode where each high-resolution full-scan in the orbitrap (*m/z* 300 to 2000, R = 120,000) was followed by high-resolution product ion scans in the orbitrap (collison-induced dissociation (CID), 35% normalized collision energy, R = 15000; higher energy collision-induced dissociation (HCD) 29% normalized collision energy ± 15 stepped collision energy, R = 15,000) within 5 s, starting with the most intense signal in the full-scan mass spectrum (isolation window 2 u). For BS^2^G-*D*_*0*_/*D*_*4*_, a charge-dependent isolation window and offset windows were employed (charge 2+: offset 1 u, isolation window 4 u; charge 3+: offset 0.66 u, isolation window 3.5 u, charge 4+: offset 0.5 u, isolation window 3 u, charge 5+: offset 0.4 u, isolation window 2.5 u, charge 6+: offset 0.33 u, isolation window 2 u, charge 7+: offset 0.28 u, isolation window 1.5 u, charge 8+: offset 0.25 u, isolation window 1 u). Dynamic exclusion (exclusion duration: 60 s, exclusion window: ±2 ppm) was enabled to allow detection of less abundant ions. Data acquisition was controlled with Xcalibur 3.0.63 (Thermo Fisher Scientific, Bremen, Germany). Peptides were identified with the Proteome Discoverer 1.4 (Thermo Fisher Scientific) using Mascot server, version 2.2. Cross-linked products were identified with the *in-house* software StavroX,[38] version 3.4.12 and MeroX version 1.4.12.[39] All cross-links were manually evaluated.

### Visualization of Cross-links

All 3D-structures were created with PyMOL (0.99rc6). Circos plots were created with Circos (0.67–7).[40]

## Results

The X-ray structure of PPAR-β/δ LBD has been determined in complex with the agonist GW0742 (pdb entry 3TKM).[36] Yet, no high-resolution structural information is available to date on how GW1516 interacts with PPAR-β/δ. Given that both agonists differ only by the presence of a fluorine atom, similar binding modes were expected for both ligands. To clarify this, we employed the chemical cross-linking/MS approach using a variety of external cross-linkers as well as the incorporation of the photo-reactive amino acid Bpa. For the latter, two Bpa variants of PPAR-β/δ LBD (amino acids 167–443) were expressed in *E*.*coli* cells using the method developed by Schultz and coworkers.[16] PPAR-β/δ LBD variants were individually transformed into *E*. *coli* cells carrying a Bpa-specific suppressor tRNA and an aminoacyl-tRNA synthetase that allows the incorporation of Bpa in place of the natural amino acid via the introduced TAG stop codon. In PPAR-β/δ LBD, either Phe-180 or Tyr-443 was replaced by the photo-reactive amino acid Bpa: Phe-180 is located on Arm II in helix 1, while Tyr-443 is located on Arm I of activation function helix 2 (AF2, helix 12) (Fig 4A). As both helices are prone to conformational changes upon ligand binding they are ideal targets for the incorporation of the photo-reactive amino acid Bpa.

### Purification of PPAR-β/δ LBD and Variants F180Bpa and Y443Bpa

PPAR-β/δ LBD and Bpa variants were purified as *N*-terminally Strep II-tagged proteins via affinity chromatography. Pure protein was obtained after TEV protease cleavage (~13 μg/g cells for PPAR-β/δ LBD and ~6.5 μg/g cells for PPAR-β/δ LBD variants). The identity of the PPAR-β/δ LBD variants and the incorporation of Bpa at the desired positions were confirmed by *in-gel* digestion and LC/MS/MS analysis.

### Cross-linking with Amine-Reactive and Zero-Length Linkers

**Fig 5:**
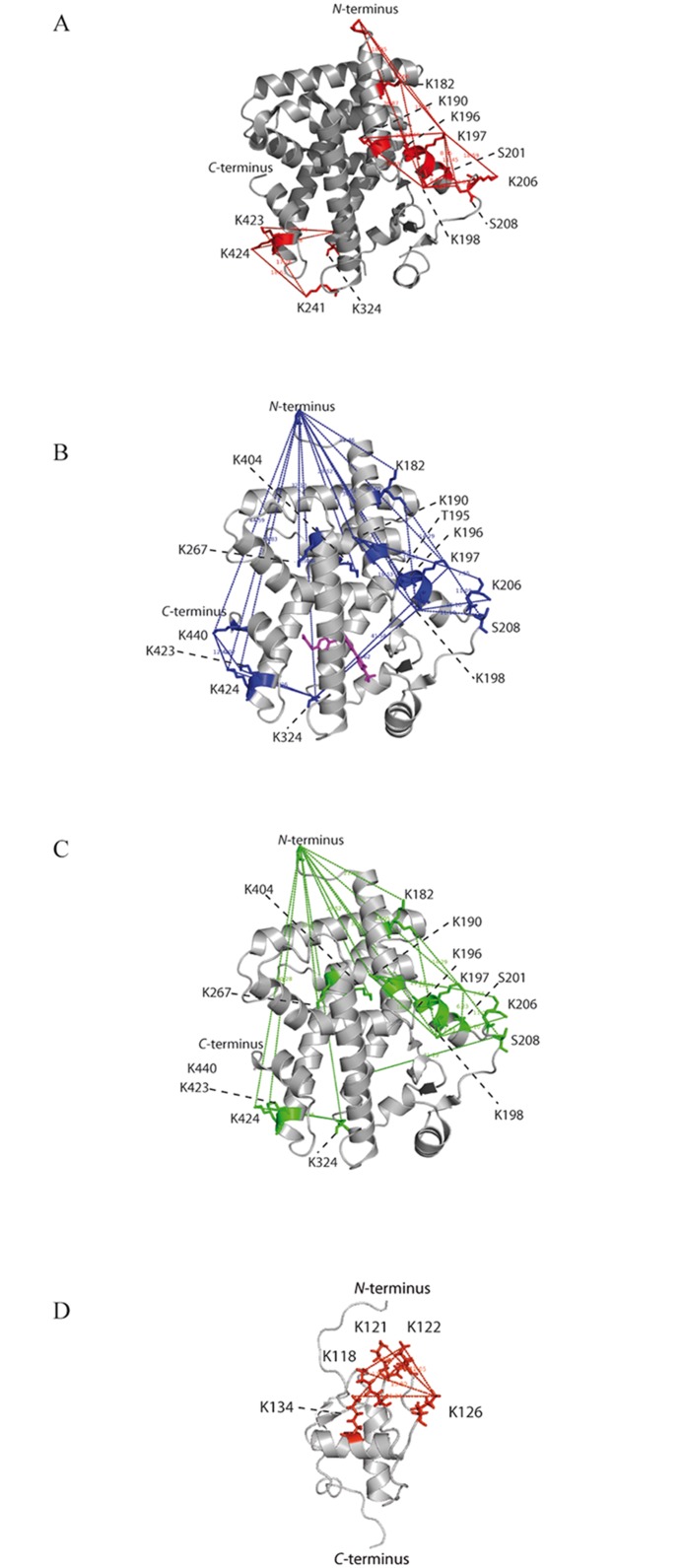
BS^2^G-cross-linked amino acids in PPAR-β/δ LBD and DBD. Cross-links identified in PPAR-β/δ in experiments with BS^2^G are mapped on the available 3D structure (3TKM). (A) PPAR-β/δ LBD without agonists, (B) PPAR-β/δ LBD with GW0742; the bound ligand is shown in magenta, (C) PPAR-β/δ LBD with GW1516, and (D) PPAR-β/δ DBD.

After purification of PPAR-β/δ LBD, we performed cross-linking experiments with the homobifunctional cross-linker BS^2^G that bridges Cα-Cα distances up to 27 Å (Fig 1A). Cross-links identified between lysines—and to a certain extent also with serines, threonines, and tyrosines—allow deducing conformational information in PPAR-β/δ. We identified a number of cross-links in the absence, but also in the presence of ligands (S1 Table). All cross-links identified in free PPAR-β/δ LBD were in accordance with the published X-ray structure (pdb entry 3TKM). Upon agonist binding, additional cross-links were identified between the *N*-terminus of the LBD (corresponding to Gly-167) and the flexible Ω-loop (for agonist GW1516) as well as the activation function helix 2 (for agonist GW0742) (Fig 4A). Interestingly, these cross-links were not identified in free PPAR-β/δ LBD and indicate conformational changes upon binding of either of the agonists. All cross-links found for BS^2^G are presented in Fig 5.

**Fig 6:**
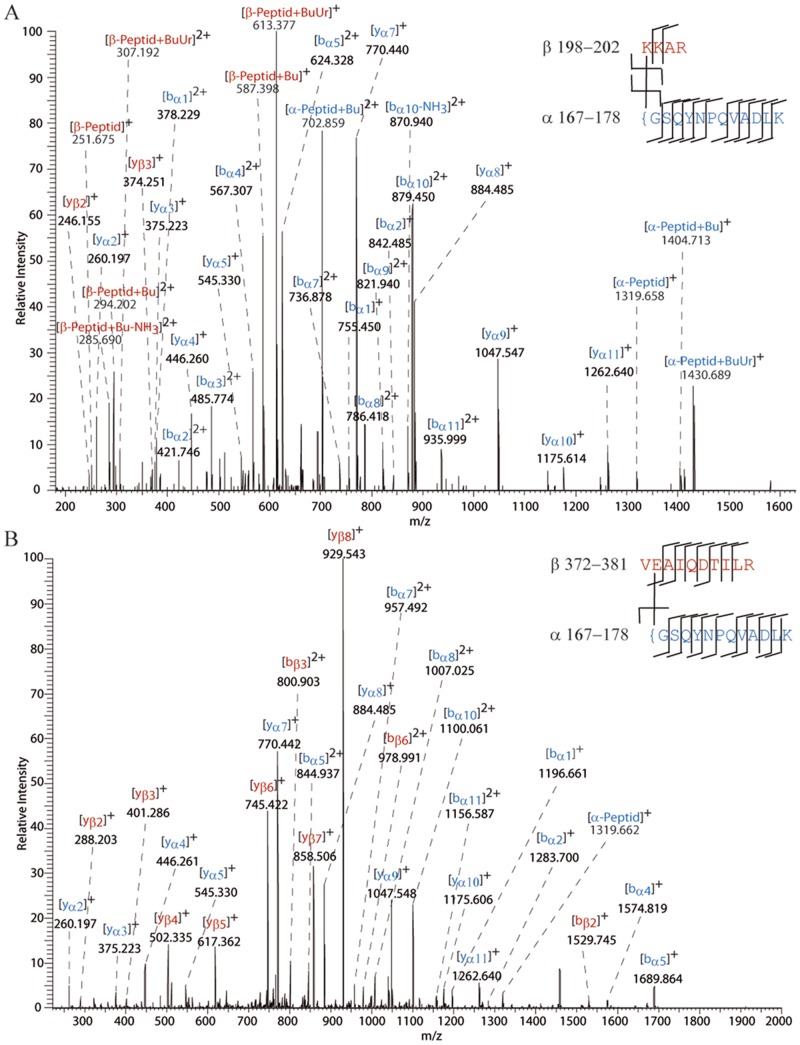
MS/MS spectra of cross-linked products in PPAR-β/δ. (A) MS/MS spectrum of a 3+ charged cross-linked product at *m/z* 673.033, identified in experiments with the urea-linker. The cross-linked product was unambiguously identified based on the presence of a y_3_ ion at *m/z* 374.251 (β-peptide) and a y_11_ ion at *m/z* 1262.640 (α-peptide). The specific fragmentation pattern of the urea-linker is described as “Bu” and “BuUr”.[15] (B) MS/MS spectrum of a 3+ charged cross-linked product at *m/z* 820.104, identified in experiments with the zero-length cross-linker DMTMM. The cross-link between the *N*-terminus of the LBD (Gly-167) and Glu-373 was identified based on a b_1_ ion at *m/z* 1196.661 (α-peptide) and a b_2_ ion at *m/z* 1283.700 (β-sequence). { denotes the *N*-terminus of the protein.

Additional cross-linking experiments were performed using the MS/MS cleavable amine-reactive urea-linker (Fig 1B) that allows an automated analysis of cross-links via its characteristic fragmentation pattern.[13, 15, 39] As such, a cross-link between the *N*-terminus of the LBD (Gly-167) and Lys-198 in PPAR-β/δ was unambiguously identified based on the specific fragmentation pattern of the urea-linker and intense backbone cleavage of the connected peptides (Fig 6A). The urea-linker is an amine-reactive homobifunctional cross-linker that bridges Cα-Cα distances up to 30 Å, which is only slightly longer than the distances BS^2^G can connect. As such, it is not surprising that for PPAR-β/δ LBD most of the cross-linking sites found in experiments with BS^2^G were also identified with the urea-linker (S2 Table).

To allow carboxylic acid side chains in PPAR-β/δ LBD to participate in the cross-linking reaction, we also employed the zero-length cross-linker DMTMM (Fig 1C). DMTMM cross-links spatially neighbored amine groups (*N*-terminus and lysine residues) and carboxyl groups (aspartic and glutamic acid residues) and thus yields complementary short-range information compared to the homobifunctional amine-reactive linkers. With DMTMM, similar cross-links were identified in PPAR-β/δ LBD in the presence as well as in the absence of ligands (S2 Table). MS/MS data of one exemplary cross-link are shown in Fig 6B. In GW0742-bound PPAR-β/δ LBD, two additional cross-links were identified, connecting the *N*-and the *C*-termini as well as the *N*-terminus (Gly167) and Glu-331 of the LBD. Both cross-links cannot be explained by the published X-ray structure. In order to be cross-linked by DMTMM, the respective amino acid side chains have to come into close spatial neighborhood indicating that PPAR-β/δ LBD adopts a specific conformation *in solution* after ligand binding.

### Photo-Cross-linking with Bpa Variants

**Fig 7:**
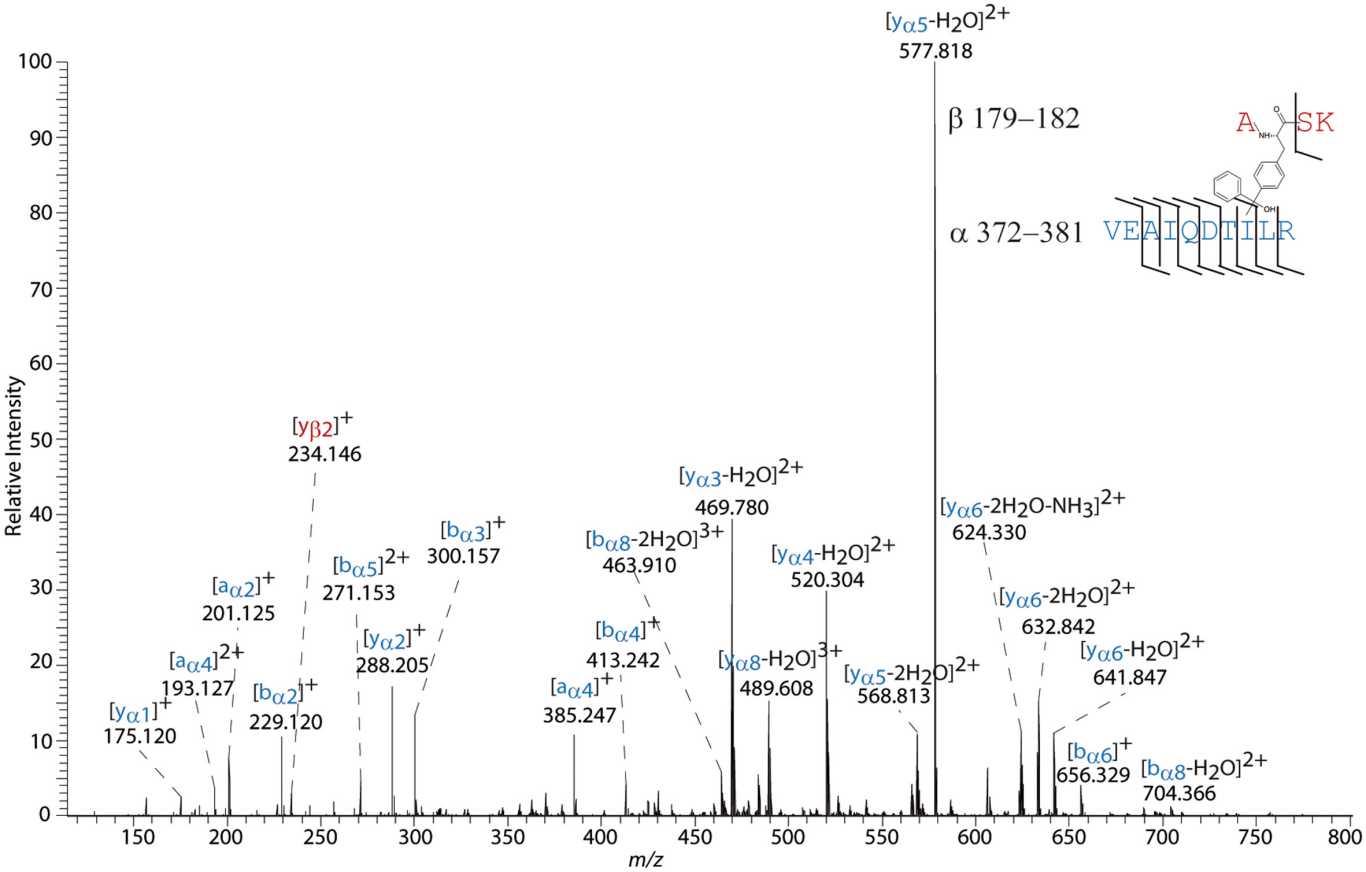
MS/MS spectrum of a 4+ charged cross-link at *m/z* 428.986, identified in photo-cross-linking experiments with the PPAR β/δ variant F180Bpa. The cross-link was unambiguously identified between Bpa-180 and Ile-379, based on the y_2_ ion at *m/z* 288.205, the y_3_ ion at *m/z* 469.780, and the b_8_ ion at *m/z* 704.366.

**Fig 8:**
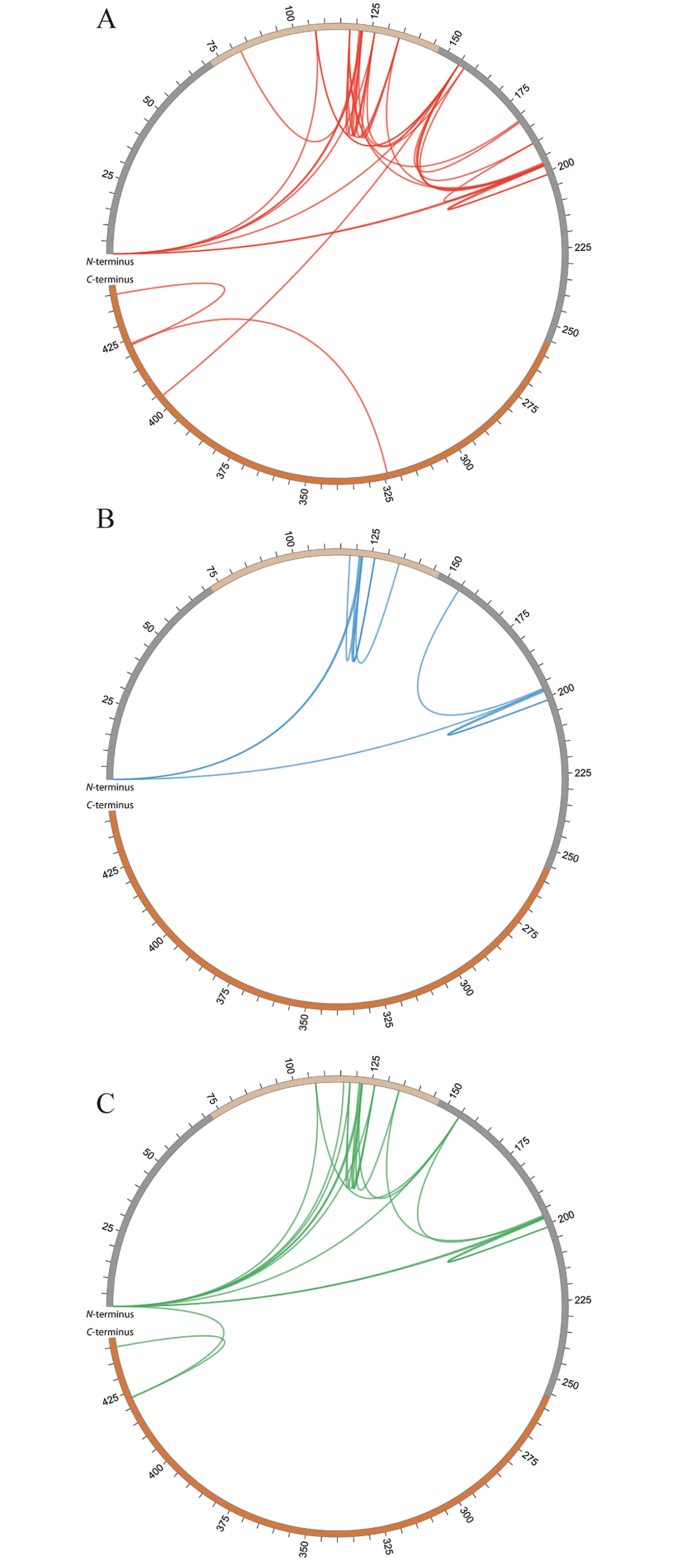
Cross-linked amino acids in full-length PPAR-β/δ. Cross-linked amino acids found with the amine-reactive linker BS^2^G are presented for full-length PPAR β/δ as Circos plots (A) without ligand, (B) with GW0742, (C) with GW1516. The flexible *N*-terminal and hinge regions are shown in grey, the LBD is shown in orange, the DBD is shown in ochre.

To obtain complementary structural information, the PPAR-β/δ LBD variants F180Bpa and Y443Bpa were produced by genetic engineering.[16] In each of the two variants, the photo-reactive amino acid Bpa was specifically introduced at a defined position in different flexible regions of PPAR-β/δ LBD to optimize monitoring conformational changes upon ligand binding (Fig 4A). Photo-cross-linking experiments were conducted in the presence and the absence of agonists GW0742 and GW1516 by irradiation with UV-A light. After the photo-cross-linking reaction, PPAR-β/δ LBD variants were enzymatically digested and the resulting peptide mixtures were analyzed by LC/ESI-MS/MS yielding several cross-links (S3 Table). For PPAR-β/δ variant F180Bpa, one cross-link was found in the absence as well as in the presence of agonists (Fig 7). For variant PPAR-β/δ Y443Bpa, two cross-links were identified in the absence, while one was identified in the presence of ligands (S3 Table). All cross-links identified with our complementary approaches are visualized as Circos plots (Fig 8A–8C, S2–S13 Figs).[40] Photo-cross-links identified in PPAR-β/δ variants F180Bpa and Y443Bpa were comparable for free and agonist-bound protein. In ligand-free PPAR-β/δ variant Y443Bpa, an additional cross-link between Bpa-443 and Ile-330 (located in the *N*-terminal region of helix 8) was identified, which perfectly matches the published X-ray structure (Fig 4A).

### Cross-linking with Full-Length PPAR-β/δ

We performed additional cross-linking experiments with full-length PPAR-β/δ comprising the DBD and the LBD (S1 Fig) to rule out that some cross-links found for PPAR-β/δ LBD might be induced by the artificially created *N*-terminus, but in fact do not represent the native structure of PPAR-β/δ. For full-length PPAR-β/δ, a high number of cross-links were identified in the flexible hinge region connecting DBD and LBD as well as in the flexible *N*-terminal region—both in the presence and in the absence of agonists (S4 Table). All unambiguously identified cross-links were in good agreement with published high-resolution 3D-structures of the DBD and the LBD. Several of these cross-links were found between the flexible *N*-terminal and hinge regions and the DBD or within the DBD (Fig 8). As such, the *N*-terminus of full-length PPAR-β/δ was cross-linked—both in the presence and in the absence of ligands—to Lys-198 in the LBD as well as to a number of amino acids in the DBD (amino acids 73–147), i.e., Lys-122, Lys-107, and Lys-126. Interestingly, only in the presence of GW1516 an additional cross-link between the *N*-terminus and Lys-423 was found (Fig 8C).

## Discussion

### Structures of PPAR-β/δ LBD

Chemical cross-linking with the amine-reactive cross-linkers BS^2^G and the urea-linker yielded a high number of cross-links in free as well as in ligand-bound PPAR-β/δ LBD (Table 1, S1 and S2 Tables). After ligand binding, several cross-links were identified between the *N*-terminus (corresponding to Gly-167) of the isolated LBD and various lysines in PPAR-β/δ LBD. Some of these cross-links cannot be explained by the X-ray structure of GW0742-bound PPAR-β/δ LBD (pdb 3TKM; Fig 5A–5C), taking the distances into account the cross-linkers can bridge (up to 30 Å). They can, however, be explained by a large degree of flexibility in the *N*-terminal region of the LBD where Gly-167 is located. As such, in the ligand-bound-state of PPAR-β/δ LBD, cross-links were identified between the *N*-terminus (Gly-167) and Lys-423/424 (*C*-terminal region of helix 11) and Lys-324 (*N*-terminal region of helix 8) (Fig 5B and 5C). With the agonist GW0742, cross-links between the *N*-terminus of the LBD (Gly-167) and Lys-440 (AF2) as well as between Lys-197/198 (helix 2) and Lys-324 (*N*-terminal region of helix 8) were identified (Fig 5B). In the GW1516-bound state, a cross-link between the *N*-terminal Gly-167 and Lys-231, located on the flexible Ω-loop, was identified (S1 Table). As no diffraction data are available for parts of the omega loop in the high-resolution X-ray structure, the location of Lys-231 is ambiguous and as such, the respective residue cannot be assigned in Fig 5C. This cross-link might be explained by a large conformational change in the Ω-loop after ligand binding, bringing the *N*-terminus of the LBD into close distance to the Ω-loop.

**Table 1:** Comparison of identified (photo)-cross-links in full-length PPAR-β/δ and PPAR-β/δ LBD. For the LBD, cross-links are summarized for all reagents used in this study, while for full-length PPAR-β/δ only BS^2^G cross-links are presented; { denotes the *N*-terminus of the protein; } denotes the *C*-terminus of the protein.

*PPAR-β/δ ligand-binding domain (LBD)*				*PPAR-β/δ full-length*			
*Cross-linked amino acids*	*Free*	*GW0742*	*GW1516*	*Cross-linked amino acids*	*Free*	*GW0742*	*GW1516*
{167+S181/K182	X	X	X	{+K107	X		X
{167+K182	X	X	X	{+S116/K118			X
{167+K190	X	X	X	{+K121/K122	X	X	X
{167+T195/K196/K197		X		{+K122	X	X	X
{167+K196/K197/K198	X	X	X	{+K126	X		X
{167+K197/K198	X	X	X	{+K155	X		X
{167+K198	X			{+K197/K198	X	X	X
{167+S201	X			{+K198	X		X
{167+K231			X	{+K423			X
{167+E264	X	X	X	K82+K121/K122	X		
{167+K267		X	X	K107+K155	X		X
{167+K324		X	X	K118+K122	X	X	X
{167+E331		X		K118+K126	X		X
{167+E373	X	X	X	K118+K134	X		
{167+D377	X	X	X	K118+S181/K182	X		
{167+K404		X	X	K118+K198	X		
{167+K423/K424		X	X	K121+K126	X	X	X
{167+K440		X		K122+K126	X	X	X
{167+}443		X		K122+K134	X	X	X
{167/G167/S168/Q169/Y170+X180	X			K122+K155			X
X180+I379	X	X	X	K122+K157	X		
S181/K182+K197/K198	X	X	X	K126+K134	X		
S181/K182+K198	X	X		K126+K155	X		
K182+K197 /K198	X	X	X	K134+K197/K198	X		X
K190+K197/K198	X	X	X	K155+K182	X		
K190+K198	X	X	X	K155+K190	X		
T195/K196/K197+K198	X	X		K155+K197	X	X	X
K197/K198+S201	X	X	X	K155+K197/K198	X		
K197/K198+K206	X	X	X	K155+K404	X		
K197/K198+S208	X	X	X	K157+K197/K198	X		
K197/K198+K206/S208/T210	X			K190+K197/K198	X		
K197/K198+K324		X		K197/K198+S201	X	X	X
K197/K198+K423/K424	X			K198+S201	X	X	X
K197+K424	X			K324+K423/K424	X		
K198+S201	X	X	X	K423+K440	X		X
K198+K206	X	X					
S201/T204/K206/S208+K302	X						
K226+Y249/K241	X						
K241+K423/K424	X						
S298/K302+K400	X						
K324+K423/K424	X	X	X				
K324+K424	X	X					
I330+X443	X						
K333+K423/K424	X						
M419+X443	X	X	X				
K423+D441	X	X	X				
K423/K424+T425/T427/T429/S430	X						
K423/K424+K440	X	X	X				

Also, in both ligand-bound states, cross-links were found between the *N*-terminal Gly-167 of the LBD and Lys-404 (*N*-terminal region of helix 11) and Lys-267 (*C*-terminal region of helix 4) (Fig 5B and 5C) that are in agreement with the distances the cross-linkers can bridge. The fact that both cross-links were not identified in free PPAR-β/δ LBD suggests the presence of a different stabilized conformation upon ligand binding.

Additional cross-linking experiments performed with the zero-length cross-linker DMTMM that delivers valuable short-range information resulted in a high number of cross-links, both in the free and in the ligand-bound state of PPAR-β/δ LBD (S2 Table). In all states, a cross-link was identified between Gly-167 and Glu-264, located in the *C*-terminal region of helix 4. Interestingly, this helix was also cross-linked by BS^2^G in the ligand-bound state (Lys-267) indicating that Gly-167 comes into close distance to helix 4 and is thus cross-linked by BS^2^G and the zero-length cross-linker DMTMM. Two additional cross-links between Gly-167 and the *C*-terminus as well as between Gly-167 and Glu-331 (located on helix 8) were identified in the GW0742-bound state. Both cross-links cannot be explained by the published X-ray structural data, again confirming the stabilization of a specific conformation where the *N*-terminus of the LBD comes into close spatial neighborhood to helices 4 and 8 (Fig 4A). Yet, with GW1516 these cross-linking sites were not identified, possibly indicating different binding modes for the two agonists GW0742 and GW1516.

The incorporation of the photo-reactive amino acid Bpa at two defined positions into PPAR-β/δ LBD, replacing either Phe-180 or Tyr-443, followed by UV-irradiation yielded only a few cross-links, underlining the selectivity of this strategy. Most of the cross-links were identified in free as well as in ligand-bound states of PPAR-β/δ LBD. For PPAR-β/δ variant F180Bpa, one cross-linking site was identified, connecting Ile-379 on helix 10 with Bpa-180 (Fig 4A). The cross-link between the Bpa-443, located on AF2, and Ile-330, located on helix 8 (Fig 4A), was found exclusively in the ligand-free state of PPAR-β/δ variant Y443Bpa. No cross-links were identified between AF2 and the flexible Ω-loop, indicating that the Ω-loop and the AF2 are not oriented towards each other, which matches the published X-ray structure (Fig 4A).

### Structures of Full-Length PPAR-β/δ

To complement our results obtained with the LBD of PPAR-β/δ, we performed cross-linking experiments with full-length PPAR-β/δ using the amine-reactive cross-linker BS^2^G. We identified a large number of cross-links in the hinge region between DBD and LBD, in the flexible *N*-terminal region, and within the DBD (Fig 8). A cross-link identified in the ligand-free state between Lys-155 (located in the hinge region) and Lys-404 (located on helix 11) of full-length PPAR-β/δ points to the same conformation as the cross-link found between Gly-167 and Lys-404 in PPAR-β/δ LBD. The fact that this cross-link was identified both in the ligand-bound state of PPAR-β/δ LBD as well as in the ligand free-state of full-length PPAR-β/δ suggests an overall presence of this specific conformation. It might be speculated that in the truncated form of PPAR-β/δ, comprising only the LBD, the *N*-terminal region and helix 1 are more flexible than in full-length PPAR-β/δ, resulting in more pronounced conformational changes upon ligand binding than in full-length PPAR β/δ. In the GW1516-bound state, a cross-link was identified between the *N*-terminus and Lys-423 (located on helix 11 in the LBD) in full-length PPAR-β/δ. This suggests that after binding of GW1516, the *N*- and *C*-termini of PPAR-β/δ come into close neigborhood (Fig 8C). The fact that this cross-link was specifically identified in GW1516-bound PPAR-β/δ, however, does not necessarily indicate that is it not present in GW0742-bound protein. Clearly, more cross-linking experiments are required to support the finding that a specific conformation is induced in full-length PPAR-β/δ only in the presence of GW1516.

Our cross-linking results yield first structural insights into the conformational changes of full-length PPAR-β/δ upon ligand binding. In the past years, crystal structures of three nuclear receptors have emerged showing interfacial coupling between the DBDs and LBDs.[41–44] Our data confirm and extend these high-resolution structural data, indicating interactions between DBD and LBD in PPAR-β/δ in solution (Fig 8). We mapped the cross-links found for full-length PPAR-β/δ into the published X-ray structure of ligand-bound PPAR-γ, co-crystallized with DNA response element, coactivator peptides, and RXR-α (pdb 3DZU) [41] (S14 Fig). All cross-links between DBD and LBD as well as those connecting the flexible hinge region with DBD and LBD in PPAR-β/δ proved to be in good agreement with the X-ray structure. One slightly longer cross-link (40.8 Å) between the hinge region and helix 11 (LBD) can easily be explained by the inherent flexibility of the hinge region. Moreover, our cross-linking data were able to deliver structural information of the highly flexible *N*-terminus of PPAR-β/δ, for which no structural data are available in the PPAR-γ structure.

Conclusively, the cross-linking/MS approach proved highly advantageous to study nuclear receptors, enabling us to reveal the interplay between DBD and LDB in PPAR-β/δ. We envision that chemical cross-linking/MS, combined with other structural, biophysical, and cell-based studies will enhance our current knowledge of how PPARs function and how conformational changes occur when activating ligands are present.

## Conclusions & Outlook

The cross-linking/MS approach revealed different conformations of PPAR-β/δ LBD and full-length PPAR-β/δ in solution, stabilizing one specific conformation through ligand binding. To our best knowledge, we performed the first studies addressing the structure of full-length PPAR-β/δ upon ligand binding, revealing the interplay between DBD and LDB in free and ligand-bound PPAR-β/δ. Our cross-linking data were able to deliver structural information also from the highly flexible *N*-terminus of PPAR-β/δ, for which no structural data are available in the PPAR-γ structure. Moreover, a close distance between the *N*- and *C*-terminal regions was observed for full-length PPAR-β/δ in the presence of GW1516. Further cross-linking experiments are planned for PPAR-β/δ in the presence of DNA response element and RXR to complement existing high-resolution 3D-structural data.

## Supporting Information

S1 Fig Amino acid sequence of PPAR-β/δ. Full-length PPAR-β/δ was subjected to cleavage with TEV protease to remove the *N*-terminal (His)_6_-tag. The TEV cleavage site is shown underlined. Due to TEV cleavage, two additional amino acids (G and A) are present at the *N*-terminus of PPAR-β/δ. The DNA-binding domain (DBD; amino acids 73–147) is highlighted in light grey; the ligand-binding domain (LBD; amino acids 167–443) in dark grey. The hinge region (amino acids 148–166; printed in italics and bold) is located between the DBD and the LBD.
(DOCX)

S2 Fig Cross-links identified with BS^2^G in ligand-free PPAR-β/δ LBD. The cross-links identified are presented as red lines; cross-linked amino acids are indicated.
(DOCX)

S3 Fig Cross-links identified with BS^2^G in GW0742-bound PPAR-β/δ LBD. The cross-links identified are presented as blue lines; cross-linked amino acids are indicated.
(DOCX)

S4 Fig Cross-links identified with BS^2^G in GW1516-bound PPAR-β/δ LBD. The cross-links identified are presented as green lines; cross-linked amino acids are indicated.
(DOCX)

S5 Fig Cross-links identified with the urea cross-linker in ligand-free PPAR-β/δ LBD. The cross-links identified are presented as red lines; cross-linked amino acids are indicated.
(DOCX)

S6 Fig Cross-links identified with the urea cross-linker in GW0742-bound PPAR-β/δ LBD. The cross-links identified are presented as blue lines; cross-linked amino acids are indicated.
(DOCX)

S7 Fig Cross-links identified with the urea cross-linker in GW1516-bound PPAR-β/δ LBD. The cross-links identified are presented as green lines; cross-linked amino acids are indicated.
(DOCX)

S8 Fig Cross-links identified with DMTMM in ligand-free PPAR-β/δ LBD. The cross-links identified are presented as red lines; cross-linked amino acids are indicated.
(DOCX)

S9 Fig Cross-links identified with DMTMM in GW0742-bound PPAR-β/δ LBD. The cross-links identified are presented as blue lines; cross-linked amino acids are indicated.
(DOCX)

S10 Fig Cross-links identified with DMTMM in GW1516-bound PPAR-β/δ LBD. The cross-links identified are presented as green lines; cross-linked amino acids are indicated.
(DOCX)

S11 Fig Photo-cross-links identified in ligand-free PPAR-β/δ variants F180Bpa and Y443Bpa. The photo-cross-links identified are presented as red lines; cross-linked amino acids are indicated.
(DOCX)

S12 Fig Photo-cross-links identified in GW0742-bound PPAR β/δ variants F180Bpa and Y443Bpa. The photo-cross-links identified are presented as blue lines; cross-linked amino acids are indicated.
(DOCX)

S13 Fig Photo-cross-links identified in GW1516-bound PPAR β/δ variants F180Bpa and Y443Bpa. The photo-cross-links identified are presented as green lines; cross-linked amino acids are indicated.
(DOCX)

S14 Fig X-ray structure of intact BVT.13-bound PPAR-γ, co-crystallized with the DNA response element (PPRE), coactivator peptides (NCOA2), and RXR-α (pdb 3DZU). Cross-links identified in full-length PPAR-β/δ are mapped in the crystal structure of PPAR-γ (shown in green). RXR-α and NCOA2 are shown in grey, PPRE in light blue, DBD of PPAR-γ in red, the hinge region in wheat, the LBD of PPAR-γ in orange, coactivator in pale cyan, and the agonist BVT.13 in magenta.
(DOCX)

S1 Table Summary of identified BS^2^G cross-links in experiments with BS^2^G in PPAR β/δ LBD. Cross-linked peptides are summarized; masses of cross-linked products with the cross-linker BS^2^G-*D*_*0*_ (light) */D*_*4*_ (heavy) are given; { denotes *N*-terminus of the protein; n denotes deamidated asparagine (corresponding to D); m denotes methionine oxidation.
(DOCX)

S2 Table Summary of identified cross-links in experiments with BS^2^G in PPAR β/δ LBD, using DMTMM or the urea-linker. Cross-linked peptides are summarized; masses of cross-linked products with the cross-linkers (DMTMM or urea-linker) are given; { denotes *N*-terminus of the protein; n denotes deamidated asparagine (corresponding to D); q denotes glutamine deamidation (corresponding to E); m denotes methionine oxidation.
(DOCX)

S3 Table Summary of identified photo-cross-links in PPAR β/δ variants F180Bpa and Y443Bpa. Photo-cross-linked peptides are summarized; masses of cross-linked products with the photo-reactive amino acid Bpa are given; { denotes *N*-terminus of the protein;} denotes *C*-terminus of the protein; q denotes glutamine deamidation (corresponding to E); m denotes methionine oxidation.
(DOCX)

S4 Table Summary of identified BS^2^G cross-links in full-length PPAR β/δ. Cross-linked peptides are summarized; masses of cross-linked products with the cross-linker BS^2^G-*D*_*0*_ (light) /*D*_*4*_ (heavy) are given; { denotes *N*-terminus of the protein;} denotes *C*-terminus of the protein; q denotes glutamine deamidation (corresponding to E); n: denotes asparagine deamidation (corresponding to D); B denotes carbamidomethylation of cysteine; m denotes methionine oxidation; X denotes Bpa.
(DOCX)

## Abbreviations

AF2: Activation function helix 2
Bpa: *Para*-benzoyl-L-phenylalanine
BS^2^G: B*is*(sulfosuccinimidyl)glutarate
CID: Collision-induced dissociation
DBD: DNA-binding domain
DMTMM: 4-(4,6-Dimethoxy-1,3,5-triazine-2-yl)-4-methylmorpholinium chloride
DTT: Dithiothreitol
ESI: Electrospray ionization
FA: Formic acid
IPTG: Isopropyl-ß-D-thiogalactopyranoside
LBD: Ligand-binding domain
LC: Liquid chromatography
MS: Mass spectrometry
MS/MS: Tandem mass spectrometry
NHS: *N*-hydroxysuccinimide
NMR: Nuclear magnetic resonance
PPAR: Peroxisome proliferator-activated receptor
TCEP: Tris(2-carboxyethyl)phosphine
TEV: Tobacco etch virus
TFA: Trifluoroacetic acid
