# Supplementary material for: Monitoring Solution Structures of Peroxisome Proliferator-Activated Receptor β/δ upon Ligand Binding
Source: PLoS One. 2016 Mar 18;11(3):e0151412. doi: 10.1371/journal.pone.0151412 (PMC4798536; doi:10.1371/journal.pone.0151412)
Supplement: S1 Fig — Full-length PPAR-β/δ was subjected to cleavage with TEV protease to remove the N-terminal (His)6-tag. The TEV cleavage site is shown underlined. Due to TEV cleavage, two additional amino acids (G and A) are present at the N-terminus of PPAR-β/δ. The DNA-binding domain (DBD; amino acids 73–147) is highlighted in light grey; the ligand-binding domain (LBD; amino acids 167–443) in dark grey. The hinge region (amino acids 148–166; printed in italics and bold) is located between the DBD and the LBD. (DOCX) [file pone.0151412.s001.docx]

MSYYHHHHHHDYDIPTTENLYFQ GAMEQPQEEA PEVREEEEKE EVAEAEGAPE LNGGPQHALP

50 60 70 80 90 100

SSSYTDLSRS SSPPSLLDQL QMGCDGASCG SLNMECRVCG DKASGFHYGV HACEGCKGFF

110 120 130 140 150 160

RRTIRMKLEY EKCERSCKIQ KKNRNKCQYC RFQKCLALGM SHNAIRF***GRM PEAEKRKLVA***

170 180 190 200 210 220

***GLTANE***GSQY NPQVADLKAF SKHIYNAYLK NFNMTKKKAR SILTGKASHT APFVIHDIET

230 240 250 260 270 280

LWQAEKGLVW KQLVNGLPPY KEISVHVFYR CQCTTVETVR ELTEFAKSIP SFSSLFLNDQ

290 300 310 320 330 340

VTLLKYGVHE AIFAMLASIV NKDGLLVANG SGFVTREFLR SLRKPFSDII EPKFEFAVKF

350 360 370 380 390 400

NALELDDSDL ALFIAAIILC GDRPGLMNVP RVEAIQDTIL RALEFHLQAN HPDAQYLFPK

410 420 430 440

LLQKMADLRQ LVTEHAQMMQ RIKKTETETS LHPLLQEIYK DMY*

**S1 Fig.** **Amino acid sequence of PPAR-β/δ.**

Full-length PPAR-β/δ was subjected to cleavage with TEV protease to remove the *N*-terminal (His)_6_-tag. The TEV cleavage site is shown underlined. Due to TEV cleavage, two additional amino acids (G and A) are present at the *N*-terminus of PPAR-β/δ. The DNA-binding domain (DBD; amino acids 73-147) is highlighted in light grey; the ligand-binding domain (LBD; amino acids 167-443) in dark grey. The hinge region (amino acids 148-166; printed in italics and bold) is located between the DBD and the LBD.
