# Supplementary material for: Monitoring Solution Structures of Peroxisome Proliferator-Activated Receptor β/δ upon Ligand Binding
Source: PLoS One. 2016 Mar 18;11(3):e0151412. doi: 10.1371/journal.pone.0151412 (PMC4798536; doi:10.1371/journal.pone.0151412)
Supplement: S9 Fig — The cross-links identified are presented as blue lines; cross-linked amino acids are indicated. (DOCX) [file pone.0151412.s009.docx]

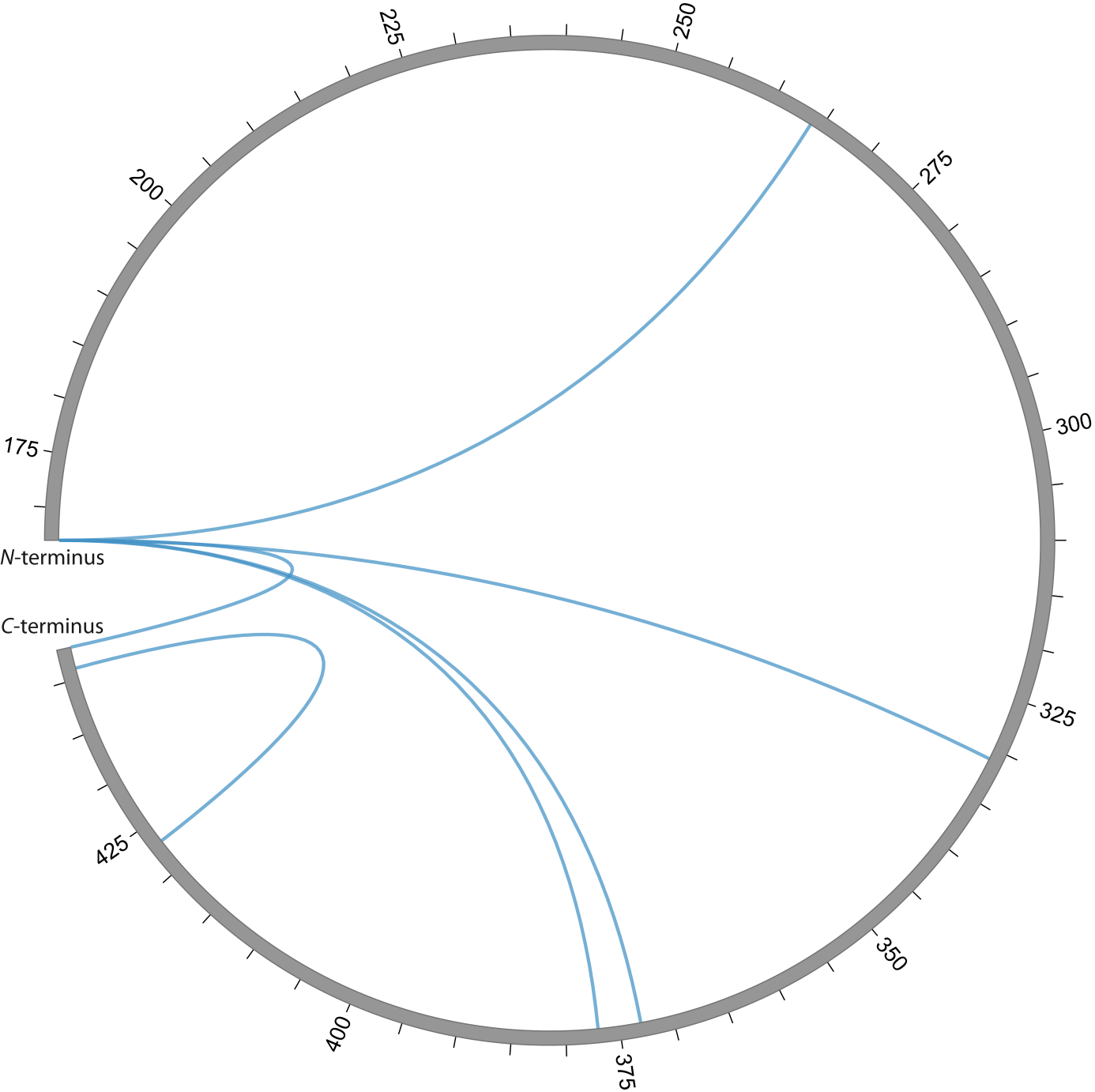


**S9 Fig. Cross-links identified with DMTMM in GW0742-bound PPAR-β/δ LBD.**

The cross-links identified are presented as blue lines; cross-linked amino acids are indicated.
