# Supplementary material for: Monitoring Solution Structures of Peroxisome Proliferator-Activated Receptor β/δ upon Ligand Binding
Source: PLoS One. 2016 Mar 18;11(3):e0151412. doi: 10.1371/journal.pone.0151412 (PMC4798536; doi:10.1371/journal.pone.0151412)
Supplement: S12 Fig — The photo-cross-links identified are presented as blue lines; cross-linked amino acids are indicated. (DOCX) [file pone.0151412.s012.docx]

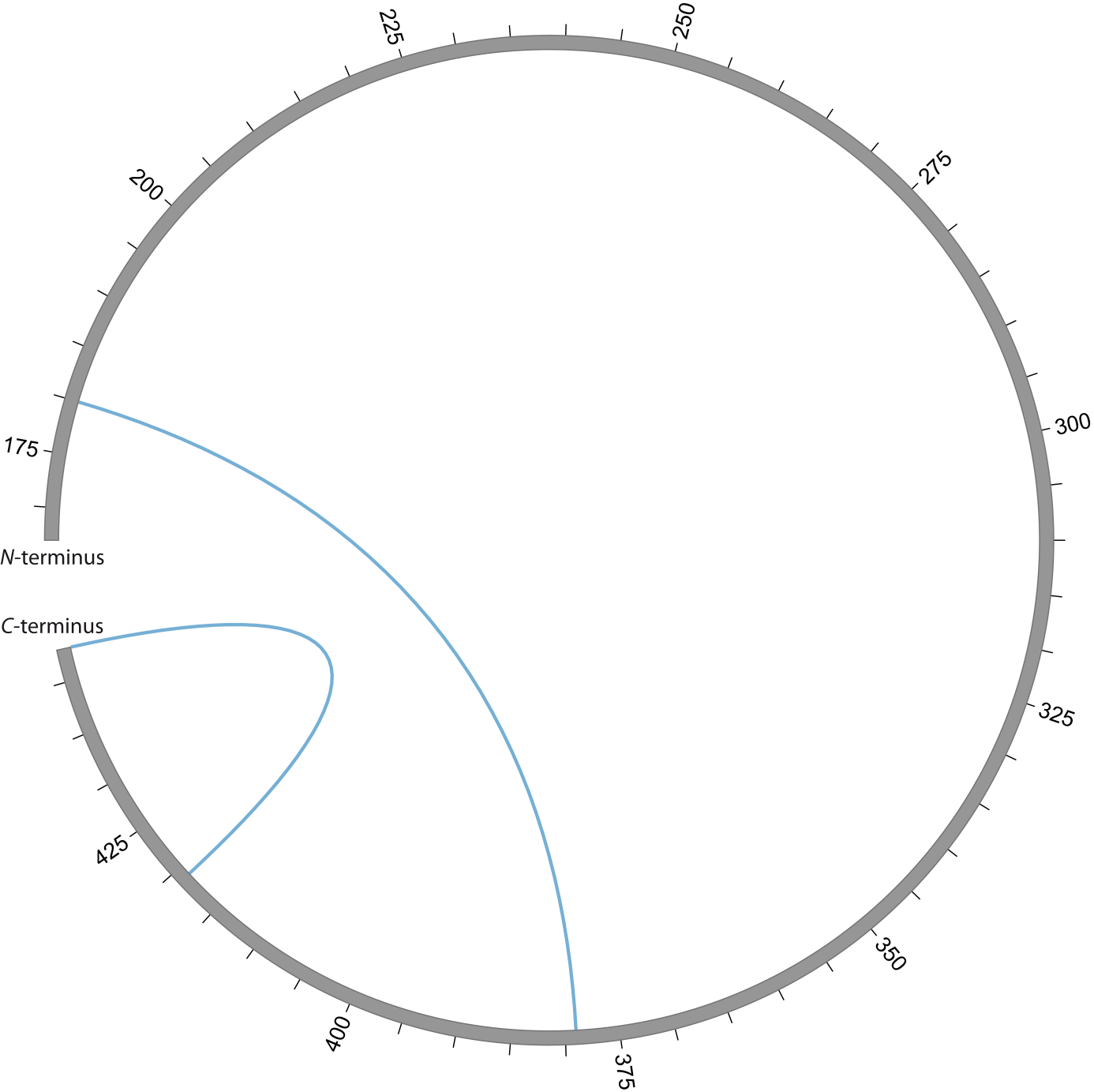


**S12 Fig. Photo-cross-links identified in GW0742-bound PPAR β/δ variants F180Bpa and Y443Bpa.**

The photo-cross-links identified are presented as blue lines; cross-linked amino acids are indicated.
