# Supplementary material for: Monitoring Solution Structures of Peroxisome Proliferator-Activated Receptor β/δ upon Ligand Binding
Source: PLoS One. 2016 Mar 18;11(3):e0151412. doi: 10.1371/journal.pone.0151412 (PMC4798536; doi:10.1371/journal.pone.0151412)
Supplement: S14 Fig — Cross-links identified in full-length PPAR-β/δ are mapped in the crystal structure of PPAR-γ (shown in green). RXR-α and NCOA2 are shown in grey, PPRE in light blue, DBD of PPAR-γ in red, the hinge region in wheat, the LBD of PPAR-γ in orange, coactivator in pale cyan, and the agonist BVT.13 in magenta. (DOCX) [file pone.0151412.s014.docx]

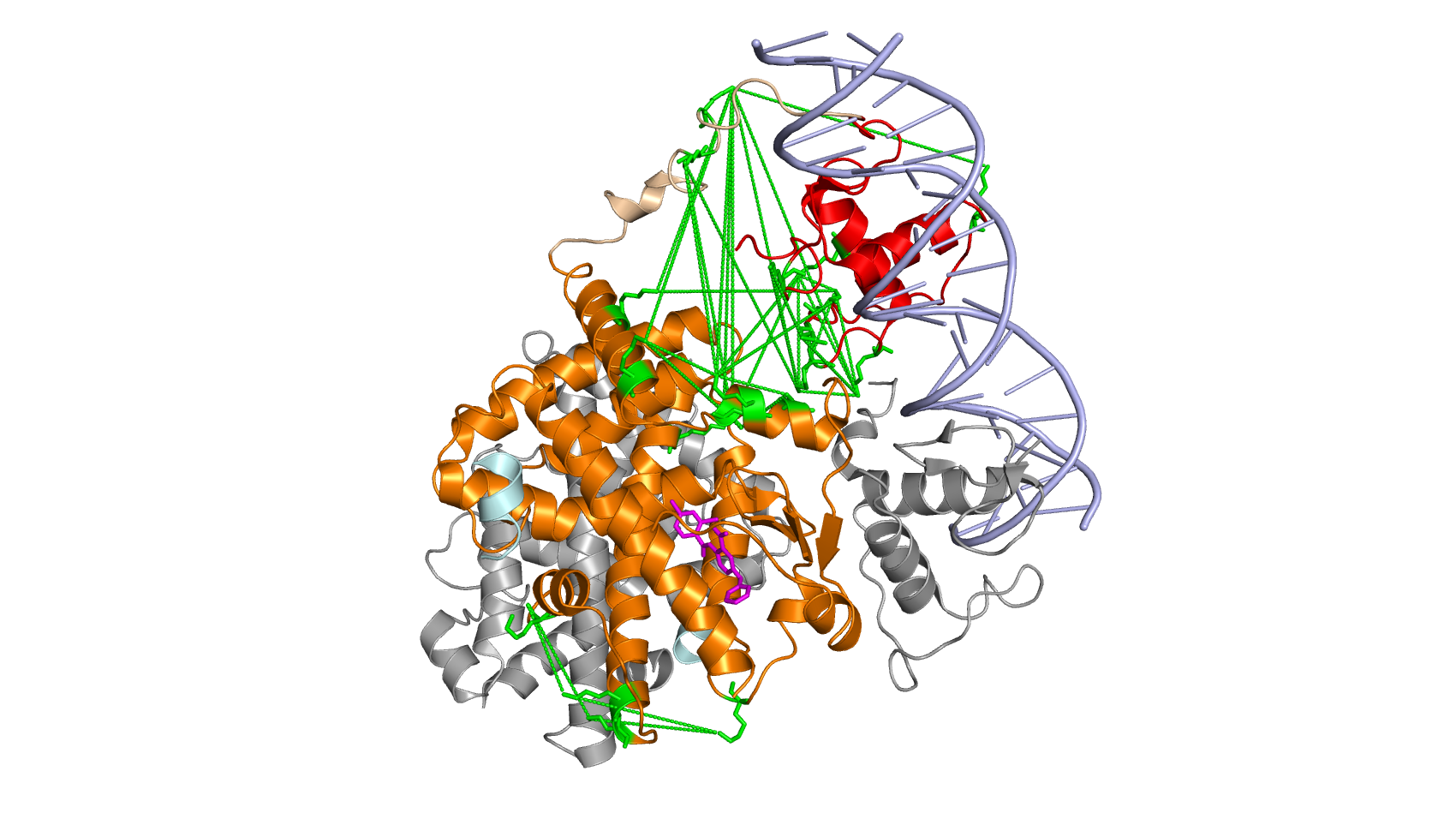


**S14 Fig. X-ray structure of intact BVT.13-bound PPAR-γ, co-crystallized with the DNA response element (PPRE), coactivator peptides (NCOA2), and RXR-α (pdb 3DZU).**

Cross-links identified in full-length PPAR-β/δ are mapped in the crystal structure of PPAR-γ (shown in green). RXR-α and NCOA2 are shown in grey, PPRE in light blue, DBD of PPAR-γ in red, the hinge region in wheat, the LBD of PPAR-γ in orange, coactivator in pale cyan, and the agonist BVT.13 in magenta.
