# Supplementary material for: Monitoring Solution Structures of Peroxisome Proliferator-Activated Receptor β/δ upon Ligand Binding
Source: PLoS One. 2016 Mar 18;11(3):e0151412. doi: 10.1371/journal.pone.0151412 (PMC4798536; doi:10.1371/journal.pone.0151412)
Supplement: S1 Table — Cross-linked peptides are summarized; masses of cross-linked products with the cross-linker BS2G-D0 (light) /D4 (heavy) are given; { denotes N-terminus of the protein; n denotes deamidated asparagine (corresponding to D); m denotes methionine oxidation. (DOCX) [file pone.0151412.s015.docx]

**S1 Table. Summary of identified BS^2^G** **cross-links in experiments with BS²G in PPAR β/δ LBD.**

Cross-linked peptides are summarized; masses of cross-linked products with the cross-linker BS^2^G-*D_0_* (light) */D_4_* (heavy) are given; { denotes *N*-terminus of the protein; n denotes deamidated asparagine (corresponding to D); m denotes methionine oxidation.

| Peptide 1 | Peptide 2 | Cross-linked  amino acids | [M+H]^+^ | *m/z* | Charge state | Cross-linker BS²G | | | Ligand | | |
| --- | --- | --- | --- | --- | --- | --- | --- | --- | --- | --- | --- |
|  |  |  |  |  |  | Light | Heavy | | Free | GW0742 | GW1516 |
| [KPFSDIIEPK]  324-333 | [KTETETSLHPLLQEIYK]  424-440 | K324+K424 | 3298.749 | 825.443 | 4 | X | |  | X | X |  |
| [IKK]  422-424 | [QLVnGLPPYKEISVHVFYR]  232-250 | K241+K423/K424 | 2743.520 | 549.510 | 5 | X | |  | X |  |  |
| [IKK]  422-424 | [QLVnGLPPYKEISVHVFYR]  232-250 | K241+K423/K424 | 2747.551 | 550.316 | 5 |  | | X | X |  |  |
| NFnMTKKK  191-198 | {GSQYNPQVADLK  {167-178 | {167+K196/K197/K198 | 2426.207 | 809.407 | 3 | X | |  | X | X |  |
| NFnMTKKK  191-198 | {GSQYNPQVADLK  {167-178 | {167+K196/K197/K198 | 2430.228 | 810.747 | 3 |  | | X | X | X | X |
| [IKK]  422-424 | KPFSDIIEPK  324-333 | K324+K423/K424 | 1656.960 | 552.992 | 3 | X | |  | X | X |  |
| [IKK]  422-424 | KPFSDIIEPK  324-333 | K324+K423/K424 | 1656.958 | 414.995 | 4 | X | |  | X | X |  |
| [IKK]  422-424 | KPFSDIIEPK  324-333 | K324+K423/K424 | 1660.983 | 554.333 | 3 |  | | X | X |  |  |
| [IKK]  422-424 | KPFSDIIEPK  324-333 | K324+K423/K424 | 1660.981 | 416.001 | 4 |  | | X | X | X | X |
| KK  197-198 | {GSQYNPQVADLK  {167-178 | {167+K197/K198 | 1689.882 | 563.966 | 3 | X | |  | X | X |  |
| KK  197-198 | {GSQYNPQVADLK  {167-178 | {167+K197/K198 | 1693.907 | 565.307 | 3 |  | | X | X | X | X |
| {GSQYNPQVADLK  {167-178 | AFSKHIYNAYLK  179-190 | {167+K182 | 2869.460 | 957.158 | 3 | X | |  | X |  |  |
| {GSQYNPQVADLK  {167-178 | AFSKHIYNAYLK  179-190 | {167+K182 | 2869.460 | 718.120 | 4 | X | |  | X |  |  |
| {GSQYNPQVADLK  {167-178 | AFSKHIYnAYLK  179-190 | {167+K182 | 2870.439 | 957.485 | 3 | X | |  | X |  |  |
| {GSQYNPQVADLK  {167-178 | AFSKHIYnAYLK  179-190 | {167+K182 | 2870.439 | 718.365 | 4 | X | |  | X |  |  |
| KK  197-198 | AFSKHIYNAYLK  179-190 | K182+K197/K198 | 1825.001 | 457.006 | 4 | X | |  | X | X |  |
| KKAR  197-200 | SILTGK  201-206 | K198+S201 | 1215.743 | 405.919 | 3 | X | |  | X |  |  |
| KKAR  197-200 | SILTGK  201-206 | K198+S201 | 1219.767 | 407.260 | 3 |  | | X | X |  |  |
| KK  197-198 | HIYNAYLKNFNMTK  183-196 | K190+K197/K198 | 2127.106 | 532.532 | 4 | X | |  | X | X |  |
| KK  197-198 | SILTGK  201-206 | K197/K198+S201 | 988.604 | 330.206 | 3 | X | |  | X |  |  |
| KK  197-198 | SILTGKASHTAPFVIHDIETLWQAEK  201-226 | K197/K198+K206 | 3262.753 | 544.632 | 6 | X | |  | X | X |  |
| KK  197-198 | SILTGKASHTAPFVIHDIETLWQAEK  201-226 | K197/K198+K206 | 3262.746 | 1088.253 | 3 | X | |  | X |  |  |
| KK  197-198 | SILTGKASHTAPFVIHDIETLWQAEK  201-226 | K197/K198+K206 | 3262.749 | 816.443 | 4 | X | |  | X |  |  |
| KK  197-198 | SILTGKASHTAPFVIHDIETLWQAEK  201-226 | K197/K198+K206 | 3262.751 | 653.356 | 5 | X | |  | X |  |  |
| KK  197-198 | ASHTAPFVIHDIETLWQAEK  207-226 | K197/K198+K208 | 2663.385 | 533.483 | 5 | X | |  | X |  |  |
| KK  197-198 | ASHTAPFVIHDIETLWQAEK  207-226 | K197/K198+K208 | 2667.409 | 667.608 | 4 |  | | X | X |  | X |
| KAR  198-200 | SILTGKASHTAPFVIHDIETLWQAEK  201-226 | K198+206 | 3361.792 | 673.164 | 5 | X | |  | X | X |  |
| KAR  198-200 | SILTGKASHTAPFVIHDIETLWQAEK  201-226 | K198+K206 | 3365.809 | 673.968 | 5 |  | | X | X | X |  |
| LLQKMADLR  401-409 | {GSQYNPQVADLK  {167-178 | {167+K404 | 2506.323 | 836.113 | 3 |  | | X |  | X | X |
| LLQKmADLR  401-409 | {GSQYNPQVADLK  {167-178 | {167+K404 | 2518.299 | 840.104 | 3 | X | |  |  | X |  |
| [IKK]  422-424 | {GSQYNPQVADLK  {167-178 | {167+K423/K424 | 1802.966 | 601.660 | 3 | X | |  |  | X |  |
| [IKK]  422-424 | {GSQYNPQVADLK  {167-178 | {167+K423/K424 | 1806.991 | 603.002 | 3 |  | | X |  | X | X |
| KAR  198-200 | HIYNAYLKNFNMTK  183-196 | K190+K198 | 2226.149 | 557.293 | 4 | X | |  |  | X |  |
| KAR  198-200 | HIYNAYLKNFNmTK  183-196 | K190+K198 | 2242.143 | 561.291 | 4 | X | |  |  | X |  |
| KK  197-198 | HIYNAYLKNFNMTK  183-196 | K190+K197/K198 | 2127.108 | 709.707 | 3 | X | |  |  | X |  |
| KK  197-198 | HIYNAYLKNFNmTK  183-196 | K190+K197/K198 | 2143.104 | 536.532 | 4 | X | |  |  | X |  |
| KK  197-198 | HIYNAYLKNFNmTK  183-196 | K190+K197/K198 | 2143.102 | 715.039 | 3 | X | |  |  | X |  |
| KK  197-198 | HIYNAYLKNFnMTK  183-196 | K190+K197/K198 | 2132.116 | 533.784 | 4 |  | | X |  | X | X |
| KK  197-198 | AFSKHIYNAYLK  179-190 | K182+ K197/K198 | 1825.001 | 365.806 | 5 | X | |  |  | X |  |
| KK  197-198 | AFSKHIYNAYLK  179-190 | K182+ K197/K198 | 1825.005 | 609.007 | 3 | X | |  |  | X |  |
| KK  197-198 | AFSKHIYNAYLK  179-190 | K182+ K197/K198 | 1829.028 | 366.611 | 5 |  | | X |  | X | X |
| KK  197-198 | AFSKHIYNAYLK  179-190 | K182+ K197/K198 | 1829.025 | 458.012 | 4 |  | | X |  | X | X |
| KK  197-198 | ASHTAPFVIHDIETLWQAEK  207-226 | K197/K198+S208 | 2663.385 | 666.602 | 4 | X | |  |  | X |  |
| {GSQYNPQVADLK  {167-179 | HIYNAYLKNFNMTK  183-196 | {167+K190 | 3175.584 | 794.652 | 4 |  | | X |  | X | X |
| {GSQYNPQVADLK  {167-179 | HIYNAYLKNFnMTK  183-196 | {167+K190 | 3176.573 | 794.899 | 4 |  | | X |  | X | X |
| {GSQYNPQVADLK  {167-179 | HIYNAYLKNFNmTK  183-196 | {167+K190 | 3191.580 | 798.650 | 4 |  | | X |  | X | X |
| {GSQYNPQVADLK  {167-179 | HIYNAYLKNFnmTK  183-196 | {167+K190 | 3192.570 | 798.898 | 4 |  | | X |  | X | X |
| {GSQYNPQVADLK  {167-179 | AFSKHIYnAYLK  179-190 | {167+K182 | 2874.460 | 719.370 | 4 |  | | X |  | X | X |
| {GSQYNPQVADLK  {167-179 | AFSKHIYnAYLK  179-190 | {167+K182 | 2874.460 | 958.825 | 3 |  | | X |  | X | X |
| {GSQYNPQVADLK  {167-179 | AFSKHIYNAYLK  179-190 | {167+K182 | 2873.478 | 958.498 | 3 |  | | X |  | X | X |
| {GSQYNPQVADLK  {167-179 | TETETSLHPLLQEIYKDMY  425-443 | {167+K440 | 3725.787 | 1242.600 | 3 | X | |  |  | X |  |
| KK  197-198 | SILTGKASHTAPFVIHDIETLWQAEK  201-226 | K197/K198+K206 | 3266.771 | 654.160 | 5 |  | | X |  | X | X |
| {GSQYNPQVADLK  {167-179 | ELTEFAKSIPSFSSLFLNDQVTLLK  261-285 | {167+K267 | 4242.170 | 1061.303 | 4 | X | |  |  | X |  |
| KK  197-198 | KPFSDIIEPK  324-333 | K197/K198+K324 | 1543.874 | 515.296 | 3 | X | |  |  | X |  |
| KPFSDIIEPK  324-333 | {GSQYNPQVADLK  {167-179 | {167+K324 | 2588.327 | 863.447 | 3 | X | |  |  | X |  |
| [IKK]  422-424 | TETETSLHPLLQEIYKDmY  425-443 | K423/K424+K440 | 2810.421 | 937.479 | 3 | X | |  |  | X |  |
| [IKK]  422-424 | TETETSLHPLLQEIYKDmY  425-443 | K423/K424+K440 | 2814.443 | 938.819 | 3 |  | | X |  | X |  |
| [NFNMTKK]  191-197 | {GSQYNPQVADLK  {167-179 | {167+T195/K196/K197 | 2301.150 | 767.722 | 3 |  | | X |  | X |  |
| LLQKmADLR  401-409 | {GSQYNPQVADLK  {167-179 | {167+K404 | 2522.324 | 841.446 | 3 |  | | X |  |  | X |
| KPFSDIIEPK  324-333 | {GSQYNPQVADLK  {167-179 | {167+K324 | 2592.353 | 864.789 | 3 |  | | X |  |  | X |
| {GSQYNPQVADLK  {167-179 | KTETETSLHPLLQEIYK  424-440 | {167+K424 | 3448.775 | 862.949 | 4 |  | | X |  |  | X |
| {GSQYNPQVADLK  {167-179 | GLVWKQLVNGLPPYK  227-241 | {167+K231 | 3130.685 | 1044.233 | 3 |  | | X |  |  | X |
| KAR  198-200 | HIYNAYLKNFNMTK  183-196 | K190+K198 | 2230.176 | 558.300 | 4 |  | | X |  |  | X |
| KK  197-198 | ASHTAPFVIHDIETLWQAEK  207-226 | K197/K198+S208 | 2667.408 | 534.287 | 5 |  | | X |  |  | X |
| KK  197-198 | HIYNAYLKNFNMTK  183-196 | K190+K197/K198 | 2131.131 | 533.538 | 4 |  | | X |  |  | X |
| KK  197-198 | HIYNAYLKNFNMTK  183-196 | K190+K197/K198 | 2131.131 | 711.049 | 3 |  | | X |  |  | X |
| KK  197-198 | HIYNAYLKNFNmTK  183-196 | K190+K197/K198 | 2147.126 | 537.537 | 4 |  | | X |  |  | X |
| KK  197-198 | HIYNAYLKNFnMTK  183-196 | K190+K197/K198 | 2128.091 | 532.778 | 4 | X | |  |  |  | X |
| KK  197-198 | SILTGKASHTAPFVIHDIETLWQAEK  201-226 | K197/K198+K206 | 3266.774 | 545.302 | 6 |  | | X |  |  | X |
| KK  197-198 | SILTGKASHTAPFVIHDIETLWQAEK  201-226 | K197/K198+K206 | 3266.775 | 817.449 | 4 |  | | X |  |  | X |
| {GSQYNPQVADLK  {167-179 | ELTEFAKSIPSFSSLFLNDQVTLLK  261-285 | {167+K267 | 4246.207 | 1062.307 | 4 |  | | X |  |  | X |
| KK  197-198 | SILTGK  201-206 | K197/K198+S201 | 992.629 | 331.548 | 3 |  | | X |  |  | X |
| KAR  198-200 | SILTGK  201-206 | K198+S201 | 1087.647 | 363.221 | 3 | X | |  |  |  | X |
| KAR  198-200 | SILTGK  201-206 | K198+S201 | 1091.672 | 364.562 | 3 |  | | X |  |  | X |
