# Supplementary material for: Monitoring Solution Structures of Peroxisome Proliferator-Activated Receptor β/δ upon Ligand Binding
Source: PLoS One. 2016 Mar 18;11(3):e0151412. doi: 10.1371/journal.pone.0151412 (PMC4798536; doi:10.1371/journal.pone.0151412)
Supplement: S2 Table — Cross-linked peptides are summarized; masses of cross-linked products with the cross-linkers (DMTMM or urea-linker) are given; { denotes N-terminus of the protein; n denotes deamidated asparagine (corresponding to D); q denotes glutamine deamidation (corresponding to E); m denotes methionine oxidation. (DOCX) [file pone.0151412.s016.docx]

**S2 Table. Summary of identified cross-links in experiments with BS²G in PPAR β/δ LBD, using DMTMM or the urea-linker.**

Cross-linked peptides are summarized; masses of cross-linked products with the cross-linkers (DMTMM or urea-linker) are given; { denotes *N*-terminus of the protein; n denotes deamidated asparagine (corresponding to D); q denotes glutamine deamidation (corresponding to E); m denotes methionine oxidation.

| Peptide 1 | Peptide 2 | Cross-linked  amino acids | [M+H]^+^ | *m/z* | Charge  state | Cross-linker | | Ligand | | |
| --- | --- | --- | --- | --- | --- | --- | --- | --- | --- | --- |
|  |  |  |  |  |  | DMTMM | Urea | Free | GW0742 | GW1516 |
| [IKK]  422-424 | [DMY}  441-443 | K423+D441 | 797.422 | 399.215 | 2 | X |  | X | X | X |
| [ELTEFAK]  261-267 | {GSQYNPQVADLK]  {167-178 | {167+E264 | 2138.078 | 713.364 | 3 | X |  | X | X | X |
| [VEAIQDTILR]  372-381 | {GSQYNPQVADLK]  {167-178 | {167+E373 | 2458.301 | 820.105 | 3 | X |  | X | X | X |
| [VEAIQDTILR]  372-381 | {GSQYNPQVADLK]  {167-178 | {167+D377 | 2458.299 | 820.104 | 3 | X |  | X | X | X |
| [VEAIQDTILR]  372-381 | {GSQYNPQVADLKAFSK]  {167-182 | {167+E373 | 2891.529 | 964.514 | 3 | X |  | X | X | X |
| [VEAIQDTILR]  372-381 | {GSQYNPQVADLKAFSK]  {167-182 | {167+D377 | 2891.528 | 964.514 | 3 | X |  | X | X | X |
| [VEAIQDTILR]  372-381 | {GSQYNPqVADLK]  {167-178 | {167+E373 | 2459.281 | 820.432 | 3 | X |  | X | X | X |
| [VEAIqDTILR]  372-381 | {GSQYNPqVADLK]  {167-178 | {167+E373 | 2460.267 | 820.760 | 3 | X |  | X | X | X |
| [VEAIQDTILR]  372-381 | {GSQYNPqVADLK]  {167-178 | {167+D377 | 2459.281 | 820.432 | 3 | X |  | X | X | X |
| [VEAIqDTILR]  372-381 | {GSQYNPQVADLKAFSK]  {167-182 | {167+E373 | 2892.515 | 964.843 | 3 | X |  | X | X | X |
| [VEAIQDTILR]  372-381 | {GSQYNPQVADLK]  {167-178 | {167+E373 | 2458.297 | 1229.652 | 2 | X |  | X | X | X |
| [VEAIQDTILR]  372-381 | {GSQYNPQVADLKAFSK]  {167-182 | {167+E373 | 2891.530 | 723.638 | 4 | X |  | X | X | X |
| [VEAIQDTILR]  372-381 | {GSQYNPQVADLK]  {167-178 | {167+E373 | 2458.297 | 615.330 | 4 | X |  |  | X |  |
| [VEAIQDTILR]  372-381 | {GSQYNPqVADLKAFSK]  {167-182 | {167+E373 | 2892.515 | 723.884 | 4 | X |  | X | X | X |
| [DMY}  441-443 | {GSQYNPQVADLK]  {167-178 | {167+}443 | 1728.791 | 864.899 | 2 | X |  |  | X |  |
| [DmY}  441-443 | {GSQYNPQVADLK]  {167-178 | {167+}443 | 1744.786 | 872.897 | 2 | X |  |  | X |  |
| [KPFSDIIEPK]  324-333 | {GSQYNPQVADLK]  {167-178 | {167+E331 | 2474.294 | 619.329 | 4 | X |  |  | X |  |
| [ELTEFAK]  261-267 | {GSQYNPQVADLK]  {167-178 | {167+E264 | 2138.075 | 1069.541 | 2 | X |  |  |  | X |
| [VEAIQDTILR]  372-381 | {GSQYNPQVADLK]  {167-178 | {167+D377 | 2458.296 | 1229.652 | 2 | X |  |  |  | X |
| [ELTEFAK]  261-267 | {GSQYNPqVADLK]  {167-178 | {167+E264 | 2139.063 | 713.693 | 3 | X |  |  |  | X |
| [KAR]  198-200 | [SILTGK]  201-206 | K198+S201 | 1187.711 | 594.359 | 2 |  | X | X | X | X |
| [KAR]  198-200 | [SILTGK]  201-206 | K198+S201 | 1187.710 | 396.575 | 3 |  | X | X | X | X |
| [KKAR]  197-200 | [SILTGK]  201-206 | K197/K198+S201 | 1315.805 | 439.273 | 3 |  | X | X | X | X |
| [KKAR]  197-200 | [SILTGK]  201-206 | K197/K198+S201 | 1315.807 | 658.407 | 2 |  | X | X | X | X |
| [KKAR]  197-200 | [SILTGK]  201-206 | K197/K198+S201 | 1315.809 | 329.708 | 4 |  | X | X | X | X |
| [KKAR]  197-200 | [AFSKHIYNAYLK]  179-190 | S181/K182+K198 | 2152.205 | 538.807 | 4 |  | X | X |  |  |
| [KKAR]  197-200 | [AFSKHIYNAYLK]  179-190 | S181/K182+K198 | 2152.204 | 718.073 | 3 |  | X | X | X |  |
| [KAR]  198-200 | [HIYNAYLKNFNMTKK]  183-197 | K190+K198 | 2454.308 | 614.332 | 4 |  | X | X |  | X |
| [KKAR]  197-200 | [HIYNAYLKNFNMTK]  183-196 | K190+K198 | 2454.310 | 614.333 | 4 |  | X | X |  | X |
| [KAR]  198-200 | [HIYNAYLKNFNMTK]  183-196 | K190+K198 | 2326.213 | 582.309 | 4 |  | X | X | X |  |
| [KK]  197-198 | [AFSKHIYNAYLK]  179-190 | S181/K182+K197/K198 | 1925.064 | 963.036 | 2 |  | X | X | X |  |
| [KK]  197-198 | [AFSKHIYNAYLK]  179-190 | S181/K182+K197/K198 | 1925.065 | 482.022 | 4 |  | X | X | X | X |
| [KK]  197-198 | [AFSKHIYNAYLK]  179-190 | S181/K182+K197/K198 | 1925.061 | 642.358 | 3 |  | X | X | X | X |
| [KK]  197-198 | [AFSKHIYnAYLK]  179-190 | S181/K182+K197/K198 | 1926.048 | 482.267 | 4 |  | X | X | X |  |
| [KK]  197-198 | [AFSKHIYnAYLK]  179-190 | S181/K182+K197/K198 | 1926.050 | 642.688 | 3 |  | X | X | X |  |
| [KK]  197-198 | [HIYNAYLKNFNMTK]  183-196 | K190+K197/K198 | 2227.169 | 743.061 | 3 |  | X | X | X | X |
| [KK]  197-198 | [HIYNAYLKNFNMTK]  183-196 | K190+K197/K198 | 2227.167 | 557.547 | 4 |  | X | X | X | X |
| [KK]  197-198 | [HIYNAYLKnFNMTK]  183-196 | K190+K197/K198 | 2228.156 | 743.390 | 3 |  | X | X | X | X |
| [KK]  197-198 | [HIYNAYLKnFNMTK]  183-196 | K190+K197/K198 | 2228.156 | 557.794 | 4 |  | X | X |  |  |
| [KK]  197-198 | [HIYNAYLKNFNmTK]  183-196 | K190+K197/K198 | 2243.165 | 748.393 | 3 |  | X | X | X | X |
| [KK]  197-198 | [HIYNAYLKNFNmTK]  183-196 | K190+K197/K198 | 2243.166 | 561.547 | 4 |  | X | X | X | X |
| [KK]  197-198 | [HIYNAYLKnFNmTK]  183-196 | K190+K197/K198 | 2244.150 | 748.722 | 3 |  | X | X |  |  |
| [KK]  197-198 | [HIYNAYLKnFNmTK]  183-196 | K190+K197/K198 | 2244.150 | 561.793 | 4 |  | X | X |  |  |
| [KK]  197-198 | [SILTGK]  201-206 | K197/K198+S201 | 1088.668 | 544.838 | 2 |  | X | X | X | X |
| [KK]  197-198 | [SILTGK]  201-206 | K197/K198+S201 | 1088.668 | 363.560 | 3 |  | X | X | X | X |
| [KK]  197-198 | {GSQYNPQVADLK]  {167-178 | {167+K197/K198 | 1789.946 | 895.476 | 2 |  | X | X | X |  |
| [KKAR]  197-200 | {GSQYNPQVADLK]  {167-178 | {167+K197/K198 | 2017.083 | 673.033 | 3 |  | X | X |  |  |
| [KKAR]  197-200 | {GSQYNPQVADLKAFSK]  {167-182 | {167+K197/K198 | 2450.320 | 817.445 | 3 |  | X | X |  |  |
| [KKAR]  197-200 | {GSQYNPQVADLKAFSK]  {167-182 | {167+K197/K198 | 2450.315 | 613.334 | 4 |  | X | X | X | X |
| [KK]  197-198 | [SILTGKASHTAPFVIHDIETLWQAEK]  201-226 | K197/K198+K206/S208/T210 | 3362.814 | 841.459 | 4 |  | X | X |  |  |
| [KK]  197-198 | [SILTGKASHTAPFVIHDIETLWQAEK]  201-226 | K197/K198+K206/S208/T210 | 3362.817 | 673.369 | 5 |  | X | X |  |  |
| [KK]  197-198 | [AFSKHIYNAYLKNFNMTK]  179-196 | S181/K182+K197/K198 | 2660.408 | 665.857 | 4 |  | X | X |  | X |
| [KK]  197-198 | [AFSKHIYNAYLKNFNMTK]  179-196 | S181/K182+K197/K198 | 2660.403 | 532.886 | 5 |  | X | X |  |  |
| [KK]  197-198 | [AFSKHIYNAYLKNFNmTK]  179-196 | S181/K182+K197/K198 | 2676.399 | 892.804 | 3 |  | X | X |  |  |
| [KK]  197-198 | [AFSKHIYNAYLKNFNmTK]  179-196 | S181/K182+K197/K198 | 2676.396 | 669.854 | 4 |  | X | X |  |  |
| [IKK]  422-424 | [KKAR]  197-200 | K197/K198+K423/K424 | 1085.715 | 543.361 | 2 |  | X | X |  |  |
| {GSQYNPQVADLK]  {167-178 | [HIYNAYLKNFNMTK]  183-196 | {167+K190 | 3271.622 | 1091.212 | 3 |  | X | X |  | X |
| {GSQYNPQVADLK]  {167-178 | [HIYNAYLKNFNmTK]  183-196 | {167+K190 | 3287.615 | 1096.543 | 3 |  | X | X |  | X |
| {GSQYNPQVADLK]  {167-178 | [HIYNAYLKNFNmTK]  183-196 | {167+K190 | 3287.619 | 822.660 | 4 |  | X | X |  | X |
| {GSQYNPQVADLK]  {167-178 | [AFSKHIYNAYLK]  179-190 | {167+S181/K182 | 2969.517 | 1485.262 | 2 |  | X | X |  |  |
| {GSQYNPQVADLK]  {167-178 | [AFSKHIYNAYLK]  179-190 | {167+S181/K182 | 2969.517 | 990.511 | 3 |  | X | X | X | X |
| {GSQYNPQVADLK]  {167-178 | [AFSKHIYnAYLK]  179-190 | {167+S181/K182 | 2970.498 | 990.838 | 3 |  | X | X |  |  |
| {GSQYNPQVADLK]  {167-178 | [HIYNAYLKNFNMTKK]  183-197 | {167+K190 | 3399.720 | 850.685 | 4 |  | X | X |  |  |
| {GSQYNPQVADLK]  {167-178 | [HIYNAYLKNFNmTKK]  183-197 | {167+K190 | 3415.712 | 854.683 | 4 |  | X | X |  |  |
| {GSQYNPQVADLK]  {167-178 | [HIYNAYLKNFnmTKK]  183-197 | {167+K190 | 3416.690 | 854.928 | 4 |  | X | X |  |  |
| [KAR]  198-200 | {GSQYNPQVADLK]  {167-178 | {167+K198 | 1888.989 | 630.335 | 3 |  | X | X |  |  |
| [SILTGKASHTAPFVIHDIETLWQAEK]  201-226 | [YGVHEAIFAMLASIVNKDGLLVANGSGFVTR]  286-316 | S201/T204/K206/S208+K302 | 6337.323 | 1268.270 | 5 |  | X | X |  |  |
| [SILTGKASHTAPFVIHDIETLWQAEK]  201-226 | [YGVHEAIFAMLASIVNKDGLLVAnGSGFVTR]  286-316 | S201/T204/K206/S208+K302 | 6338.301 | 1268.466 | 5 |  | X | X |  |  |
| [ALEFHLQANHPDAQYLFPKLLQK]  382-404 | [YGVHEAIFAMLASIVNKDGLLVANGSGFVTR]  286-316 | S298/K302+K400 | 6166.242 | 1234.054 | 5 |  | X | X |  |  |
| [ALEFHLQANHPDAQYLFPKLLQK]  382-404 | [YGVHEAIFAMLASIVNKDGLLVAnGSGFVTR]  286-316 | S298/K302+K400 | 6167.224 | 1234.251 | 5 |  | X | X |  |  |
| [SILTGK]  201-206 | {GSQYNPQVADLK]  {167-178 | {167+S201 | 2566.357 | 856.124 | 3 |  | X | X |  |  |
| [KAR]  198-200 | [NFNMTKK]  191-197 | T195/K196/K197+K198 | 1451.780 | 726.393 | 2 |  | X | X | X |  |
| [KAR]  198-200 | [NFNMTKK]  191-197 | T195/K196/K197+K198 | 1451.779 | 484.598 | 3 |  | X | X | X |  |
| [KAR]  198-200 | [NFNMTKK]  191-197 | T195/K196/K197+K198 | 1451.778 | 363.700 | 4 |  | X | X | X |  |
| [KAR]  198-200 | [NFNmTKK]  191-197 | T195/K196/K197+K198 | 1467.777 | 734.392 | 2 |  | X | X |  |  |
| [KAR]  198-200 | [NFNmTKK]  191-197 | T195/K196/K197+K198 | 1467.773 | 367.699 | 4 |  | X | X |  |  |
| [KK]  197-198 | {GSQYNPQVADLKAFSK]  {167-182 | {167+K197/K198 | 2223.176 | 556.550 | 4 |  | X | X |  | X |
| [KAR]  198-200 | {GSQYNPQVADLKAFSK]  {167-182 | {167+K198 | 2322.223 | 581.311 | 4 |  | X | X |  |  |
| [IKK]  422-424 | [KKAR]  197-200 | K197+K424 | 1085.715 | 362.567 | 3 |  | X | X |  |  |
| [IKK]  422-424 | [KPFSDIIEPK]  324-333 | K324+K423/K424 | 1757.021 | 878.993 | 2 |  | X | X |  |  |
| [IKK]  422-424 | [KPFSDIIEPK]  324-333 | K324+K423/K424 | 1757.021 | 586.330 | 3 |  | X | X |  |  |
| [IKK]  422-424 | [KPFSDIIEPK]  324-333 | K324+K423/K424 | 1757.021 | 439.998 | 4 |  | X | X |  |  |
| [IKK]  422-424 | [TETETSLHPLLQEIYKDmY}  425-443 | K423/K424+K440 | 2910.481 | 970.810 | 3 |  | X | X | X | X |
| [IKK]  422-424 | [TETETSLHPLLQEIYK}  425-440 | K423/K424+T425/T427/T429/S430 | 2485.355 | 829.105 | 3 |  | X | X |  |  |
| [IKK]  422-424 | [KPFSDIIEPKFEFAVK]  324-339 | K333+K423/K424 | 2478.339 | 620.340 | 4 |  | X | X |  |  |
| [QLVNGLPPYKEISVHVFYR]  232-250 | [ASHTAPFVIHDIETLWQAEKGLVWK]  207-231 | K226+Y240/K241 | 5330.804 | 1066.967 | 5 |  | X | X |  |  |
| [QLVNGLPPYKEISVHVFYR]  232-250 | [ASHTAPFVIHDIETLWqAEKGLVWK]  207-231 | K226+Y240/K241 | 5331.811 | 1067.168 | 5 |  | X | X |  |  |
| [KAR]  198-200 | [NFNmTKK]  191-197 | T195/K196/K197+K198 | 1467.774 | 489.916 | 3 |  | X |  |  |  |
| [KAR]  198-200 | [AFSKHIYNAYLK]  179-190 | S181/K182+K198 | 2024.108 | 675.358 | 3 |  | X |  | X |  |
| {GSQYNPQVADLKAFSK]  {167-182 | [HIYNAYLKNFNMTKK]  183-197 | {167+K190 | 3832.954 | 767.397 | 5 |  | X |  |  | X |
| {GSQYNPQVADLK]  {167-178 | [AFSKHIYNAYLKNFNMTK]  179-196 | {167+K190 | 3704.855 | 926.969 | 4 |  | X |  |  | X |
| {GSQYNPQVADLKAFSK]  {167-182 | [HIYNAYLKNFNMTK]  183-196 | {167+K190 | 3704.853 | 741.776 | 5 |  | X |  |  | X |
| {GSQYNPQVADLKAFSK]  {167-182 | [HIYNAYLKNFNMTK]  183-196 | {167+K190 | 3704.845 | 1235.620 | 3 |  | X |  |  | X |
| [KK]  197-198 | [AFSKHIYNAYLKNFNMTK]  179-196 | S181/K182+K197/K198 | 2660.407 | 887.474 | 3 |  | X |  |  | X |
| {GSQYNPQVADLKAFSK]  {167-182 | [HIYNAYLKNFNmTKK]  183-197 | {167+K190 | 3848.946 | 770.595 | 5 |  | X |  |  | X |
| [KK]  197-198 | {GSQYNPQVADLKAFSK]  {167-182 | {167+K197/K198 | 2223.180 | 741.731 | 3 |  | X |  |  | X |
| {GSQYNPQVADLK]  {167-178 | [AFSKHIYNAYLKNFNmTK]  179-196 | {167+K190 | 3720.968 | 930.968 | 4 |  | X |  |  | X |
