# Supplementary material for: Monitoring Solution Structures of Peroxisome Proliferator-Activated Receptor β/δ upon Ligand Binding
Source: PLoS One. 2016 Mar 18;11(3):e0151412. doi: 10.1371/journal.pone.0151412 (PMC4798536; doi:10.1371/journal.pone.0151412)
Supplement: S3 Table — Photo-cross-linked peptides are summarized; masses of cross-linked products with the photo-reactive amino acid Bpa are given; { denotes N-terminus of the protein;} denotes C-terminus of the protein; q denotes glutamine deamidation (corresponding to E); m denotes methionine oxidation. (DOCX) [file pone.0151412.s017.docx]

**S3 Table. Summary of identified photo-cross-links in PPAR β/δ variants F180Bpa and Y443Bpa.**

Photo-cross-linked peptides are summarized; masses of cross-linked products with the photo-reactive amino acid Bpa are given; { denotes *N*-terminus of the protein; } denotes *C*-terminus of the protein; q denotes glutamine deamidation (corresponding to E); m denotes methionine oxidation.

| Peptide 1 | Peptide 2 | Cross-linked  amino acids | [M+H]^+^ | *m/z* | Charge  state | Variant F180Bpa | | Ligand | | |
| --- | --- | --- | --- | --- | --- | --- | --- | --- | --- | --- |
|  |  |  |  |  |  | Without water loss | With water loss | Free | GW0742 | GW1516 |
| [AXSK]  179-182 | [VEAIQDTILR]  372-381 | X180+I379 | 1712.923 | 571.646 | 3 | X |  | X | X | X |
| [AXSK]  179-182 | [VEAIQDTILR]  372-381 | X180+I379 | 1712.923 | 428.986 | 4 | X |  | X | X | X |
| [AXSK]  179-182 | [VEAIQDTILR]  372-381 | X180+I379 | 1712.923 | 856.965 | 2 | X |  | X | X | X |
| [AXSK]  179-182 | [VEAIqDTILR]  372-381 | X180+I379 | 1713.904 | 571.973 | 3 | X |  | X |  |  |
| [AXSK]  179-182 | [VEAIqDTILR]  372-381 | X180+I379 | 1713.905 | 429.232 | 4 | X |  | X | X |  |
| [VEAIQDTILR]  372-381 | {GSQYNPQVADLKAXSK]  {167-182 | X180+I379 | 3013.562 | 754.146 | 4 | X |  | X | X | X |
| [VEAIqDTILR]  372-381 | {GSQYNPQVADLKAXSK]  {167-182 | X180+I379 | 3014.555 | 1005.523 | 3 | X |  | X |  | X |
| [AXSK]  179-182 | {GSQYNPQVADLK]  {167-178 | X180+{167/G167/S168/Q169/Y170 | 1874.925 | 625.647 | 3 | X |  | X |  |  |
| [AXSK]  179-182 | [VEAIQDTILR]  372-381 | X180+I379 | 1694.912 | 856.965 | 2 |  | X | X | X | X |
| [AXSK]  179-182 | [VEAIQDTILR]  372-381 | X180+I379 | 1694.911 | 424.483 | 4 |  | X | X | X | X |
| [AXSK]  179-182 | [VEAIqDTILR]  372-381 | X180+I379 | 1695.894 | 424.729 | 4 |  | X | X | X | X |
| [VEAIQDTILR]  372-381 | {GSQYNPQVADLKAXSK]  {167-182 | X180+I379 | 2995.553 | 999.189 | 3 |  | X | X |  | X |
| [VEAIQDTILR]  372-381 | {GSQYNPQVADLKAXSK]  {167-182 | X180+I379 | 3013.569 | 1005.194 | 3 | X |  | X | X | X |
| [VEAIQDTILR]  372-381 | {GSQYNPQVADLKAXSK]  {167-182 | X180+I379 | 2995.554 | 749.644 | 4 |  | X | X |  | X |
| [VEAIqDTILR]  372-381 | {GSQYNPQVADLKAXSK]  {167-182 | X180+I379 | 2996.543 | 999.519 | 3 |  | X |  |  | X |
| [AXSK]  179-182 | [VEAIQDTILR]  372-381 | X180+I379 | 1694.912 | 565.642 | 3 |  | X |  | X |  |
| [VEAIQDTILR]  372-381 | {GSQYNPQVADLKAXSK]  {167-182 | X180+I379 | 3013.565 | 603.519 | 5 | X |  | X |  |  |
| [VEAIQDTILR]  372-381 | {GSQYNPQVADLKAXSK]  {167-182 | X180+I379 | 2995.554 | 599.917 | 5 |  | X | X |  |  |
| Peptide 1 | Peptide 2 | Cross-linked amino acids | [M+H]^+^ | *m/z* | Charge state | Variant Y443Bpa | | Ligand | | |
|  |  |  |  |  |  | Without water loss | With water loss | Free | GW0742 | GW1516 |
| [DMX}  441-443 | [QLVTEHAQMMQR]  410-421 | M419+X443 | 1986.889 | 497.478 | 4 | X |  | X |  |  |
| [DMX}  441-443 | [QLVTEHAQMMQR]  410-421 | M419+X443 | 1986.889 | 662.968 | 3 | X |  | X |  |  |
| [DMX}  441-443 | [QLVTEHAQMMQR]  410-421 | M419+X443 | 1968.881 | 656.965 | 3 |  | X | X |  |  |
| [DMX}  441-443 | [QLVTEHAQMMQR]  410-421 | M419+X443 | 1968.874 | 492.974 | 4 |  | X | X |  |  |
| [DMX}  441-443 | [KPFSDIIEPK]  324-333 | I330+X443 | 1688.824 | 563.609 | 3 | X |  | X |  |  |
| [DMX}  441-443 | [KPFSDIIEPK]  324-333 | I330+X443 | 1688.824 | 422.957 | 4 | X |  | X |  |  |
| [DMX}  441-443 | [HAQMMQR]  415-421 | M419+X443 | 1416.586 | 472.860 | 3 | X |  | X | X |  |
| [HAQMMQR]  415-421 | [IYKDMX}  438-443 | M419+X443 | 1820.828 | 455.956 | 4 | X |  | X | X | X |
| [HAQMMQR]  415-421 | [IYKDMX}  438-443 | M419+X443 | 1820.828 | 607.607 | 3 | X |  | X |  |  |
| [DMX}  441-443 | [QLVTEHAQMmQR]  410-421 | M419+X443 | 1984.871 | 662.291 | 3 |  | X |  | X |  |
| [HAQMmQR]  415-421 | [IYKDMX}  438-443 | M419+X443 | 1836.823 | 459.955 | 4 | X |  |  | X | X |
| [HAQMmQR]  415-421 | [IYKDmX}  438-443 | M419+X443 | 1834.807 | 459.451 | 4 |  | X |  | X | X |
| [DMX}  441-443 | [HAQMmQR]  415-421 | M419+X443 | 1432.581 | 478.192 | 3 | X |  |  | X | X |
| [DMX}  441-443 | [HAQMmQR]  415-421 | M419+X443 | 1414.570 | 472.189 | 3 |  | X |  | X | X |
| [DMX}  441-443 | [HAQMmQR]  415-421 | M419+X443 | 1414.570 | 707.782 | 2 |  | X |  | X | X |
| [HAQMmQR]  415-421 | [IYKDMX}  438-443 | M419+X443 | 1818.812 | 455.453 | 4 |  | X |  |  | X |
