# Supplementary material for: Monitoring Solution Structures of Peroxisome Proliferator-Activated Receptor β/δ upon Ligand Binding
Source: PLoS One. 2016 Mar 18;11(3):e0151412. doi: 10.1371/journal.pone.0151412 (PMC4798536; doi:10.1371/journal.pone.0151412)
Supplement: S4 Table — Cross-linked peptides are summarized; masses of cross-linked products with the cross-linker BS2G-D0 (light) /D4 (heavy) are given; { denotes N-terminus of the protein;} denotes C-terminus of the protein; q denotes glutamine deamidation (corresponding to E); n: denotes asparagine deamidation (corresponding to D); B denotes carbamidomethylation of cysteine; m denotes methionine oxidation; X denotes Bpa. (DOCX) [file pone.0151412.s018.docx]

**S4 Table. Summary of identified BS^2^G** **cross-links in full-length PPAR β/δ.**

Cross-linked peptides are summarized; masses of cross-linked products with the cross-linker BS^2^G-*D_0_* (light) */D_4_* (heavy) are given; { denotes *N*-terminus of the protein; } denotes *C*-terminus of the protein; q denotes glutamine deamidation (corresponding to E); n: denotes asparagine deamidation (corresponding to D); B denotes carbamidomethylation of cysteine; m denotes methionine oxidation; X denotes Bpa.

| Peptide 1 | Peptide 2 | Cross-linked amino acids | [M+H]^+^ | *m/z* | Charge state | Cross-linker BS²G | | Ligand | | |
| --- | --- | --- | --- | --- | --- | --- | --- | --- | --- | --- |
|  |  |  |  |  |  | Light | Heavy | Free | GW0742 | GW1516 |
| [NKBQYBR] 125-131 | [FQKBLALGMSHNAIR]  132-146 | K126+K134 | 2869.349 | 718.093 | 4 | X |  | X |  |  |
| [NKBQYBR] 125-131 | [FQKBLALGMSHNAIR]  132-146 | K126+K134 | 2873.366 | 958.46 | 3 |  | X | X |  |  |
| [NKBQYBR] 125-131 | [FQKBLALGmSHNAIR]  132-146 | K126+K134 | 2889.368 | 578.679 | 5 |  | X | X |  |  |
| [NKBQYBR] 125-131 | [FQKBLALGMSHnAIR]  132-146 | K126+K134 | 2874.351 | 719.343 | 4 |  | X | X |  |  |
| [KK]  197-198 | [KLVAGLTANEGSQYNPQVADLK]  157-178 | K157+K197/K198 | 2686.441 | 896.152 | 3 | X |  | X |  |  |
| [KK]  197-198 | [KLVAGLTANEGSQYNPQVADLK]  157-178 | K157+K197/K198 | 2690.462 | 673.371 | 4 |  | X | X |  |  |
| [SBKIQK]  116-121 | [FQKBLALGMSHNAIR]  132-146 | K118+K134 | 2608.347 | 652.842 | 4 |  | X | X |  |  |
| [SBKIQK]  116-121 | [FQKBLALGMSHNAIR]  132-146 | K118+K134 | 2608.350 | 870.121 | 3 |  | X | X |  |  |
| [SBKIqK]  116-121 | [FQKBLALGMSHNAIR]  132-146 | K118+K134 | 2605.304 | 652.081 | 4 | X |  | X |  |  |
| [mPEAEKR]  150-156 | [AFSKHIYNAYLK]  179-190 | K155+K182 | 2430.243 | 810.752 | 3 |  | X | X |  |  |
| [MPEAEKR]  150-156 | [AFSKHIYNAYLK]  179-190 | K155+K182 | 2414.247 | 805.421 | 3 |  | X | X |  |  |
| [MPEAEKR]  150-156 | [AFSKHIYNAYLK]  179-190 | K155+K182 | 2410.224 | 804.080 | 3 | X |  | X |  |  |
| [MPEAEKR]  150-156 | [AFSKHIYNAYLK]  179-190 | K155+K182 | 2410.224 | 603.312 | 4 | X |  | X |  |  |
| [SBKIQK]  116-121 | [AFSKHIYNAYLK]  179-190 | K118+S181/K182 | 2317.235 | 580.064 | 4 |  | X | X |  |  |
| [SBKIQK]  116-121 | [AFSKHIYNAYLK]  179-190 | K118+S181/K182 | 2317.233 | 773.082 | 3 |  | X | X |  |  |
| [KK]  197-198 | [HIYNAYLKNFNMTK]  183-196 | K190+K197/K198 | 2131.131 | 533.538 | 4 |  | X | X |  |  |
| [KK]  197-198 | [HIYNAYLKNFNmTK]  183-196 | K190+K197/K198 | 2143.101 | 536.531 | 4 | X |  | X |  |  |
| [KK]  197-198 | [FQKBLALGMSHNAIR]  132-146 | K134+K197/K198 | 2120.138 | 530.790 | 4 |  | X | X |  |  |
| [MPEAEKR]  150-156 | [NKBQYBR]  125-131 | K126+K155 | 1987.910 | 663.308 | 3 |  | X | X |  |  |
| [MPEAEKR]  150-156 | [MKLEYEK]  106-112 | K107+K155 | 1899.948 | 475.742 | 4 |  | X | X |  | X |
| [MPEAEKR]  150-156 | [MKLEYEK]  106-112 | K107+K155 | 1899.950 | 950.479 | 2 |  | X | X |  |  |
| [MPEAEKR]  150-156 | [MKLEYEK]  106-112 | K107+K155 | 1895.927 | 632.647 | 3 | X |  | X |  | X |
| [MPEAEKR]  150-156 | [MKLEYEK]  106-112 | K107+K155 | 1895.924 | 474.736 | 4 | X |  | X |  | X |
| [MPEAEKR]  150-156 | [MKLEYEK]  106-112 | K107+K155 | 1895.927 | 948.467 | 2 | X |  | X |  |  |
| [MPEAEKR]  150-156 | [MKLEYEK]  106-112 | K107+K155 | 1899.949 | 633.988 | 3 |  | X | X |  | X |
| [MPEAEKR]  150-156 | [mKLEYEK]  106-112 | K107+K155 | 1911.920 | 478.735 | 4 | X |  | X |  |  |
| [mPEAEKR]  150-156 | [MKLEYEK]  106-112 | K107+K155 | 1911.919 | 637.978 | 3 | X |  | X |  |  |
| [mPEAEKR]  150-156 | [MKLEYEK]  106-112 | K107+K155 | 1915.947 | 639.320 | 3 |  | X | X |  | X |
| [mPEAEKR]  150-156 | [MKLEYEK]  106-112 | K107+K155 | 1911.922 | 956.465 | 2 | X |  | X |  |  |
| [mPEAEKR]  150-156 | [mKLEYEK]  106-112 | K107+K155 | 1931.939 | 483.741 | 4 |  | X | X |  | X |
| [mPEAEKR]  150-156 | [mKLEYEK]  106-112 | K107+K155 | 1927.915 | 482.734 | 4 | X |  | X |  | X |
| [mPEAEKR]  150-156 | [mKLEYEK]  106-112 | K107+K155 | 1931.940 | 644.652 | 3 |  | X | X |  |  |
| [mPEAEKR]  150-156 | [mKLEYEK]  106-112 | K107+K155 | 1927.915 | 643.310 | 3 | X |  | X |  | X |
| [SBKIQK]  116-121 | [NKBQYBR]  125-131 | K118+K126 | 1886.869 | 629.628 | 3 | X |  | X |  | X |
| [SBKIQK]  116-121 | [NKBQYBR]  125-131 | K118+K126 | 1890.892 | 630.967 | 3 |  | X | X |  | X |
| [IKK]  422-424 | [KPFSDIIEPK]  324-333 | K324+K423/K424 | 1660.982 | 554.332 | 3 |  | X | X |  |  |
| [IKK]  422-424 | [KPFSDIIEPK]  324-333 | K324+K423/K424 | 1656.959 | 552.991 | 3 | X |  | X |  |  |
| [IQKK]  119-122 | [NKBQYBR]  125-131 | K121+K126 | 1639.804 | 547.273 | 3 | X |  | X | X | X |
| [IQKK]  119-122 | [NKBQYBR]  125-131 | K121+K126 | 1639.803 | 410.706 | 4 | X |  | X | X | X |
| [IQKK]  119-122 | [NKBQYBR]  125-131 | K121+K126 | 1643.828 | 548.614 | 3 |  | X | X | X | X |
| [IQKK]  119-122 | [NKBQYBR]  125-131 | K121+K126 | 1643.828 | 411.712 | 4 |  | X | X | X | X |
| [IQKK]  119-122 | [nKBQYBR]  125-131 | K121+K126 | 1640.787 | 410.952 | 4 | X |  | X | X | X |
| [IQKK]  119-122 | [nKBQYBR]  125-131 | K121+K126 | 1640.788 | 547.601 | 3 | X |  | X | X | X |
| [IQKK]  119-122 | [nKBQYBR]  125-131 | K121+K126 | 1644.813 | 411.959 | 4 |  | X | X | X | X |
| [IQKK]  119-122 | [nKBQYBR]  125-131 | K121+K126 | 1644.815 | 548.943 | 3 |  | X | X | X | X |
| [KNR]  122-124 | [NKBQYBR]  125-131 | K122+K126 | 1540.713 | 514.243 | 3 | X |  | X | X | X |
| [KNR]  122-124 | [NKBQYBR]  125-131 | K122+K126 | 1540.712 | 385.934 | 4 | X |  | X | X | X |
| [KNR]  122-124 | [NKBQYBR]  125-131 | K122+K126 | 1544.736 | 386.939 | 4 |  | X | X | X | X |
| [KNR]  122-124 | [nKBQYBR]  125-131 | K122+K126 | 1541.695 | 386.179 | 4 | X |  | X | X | X |
| [KNR]  122-124 | [nKBQYBR]  125-131 | K122+K126 | 1545.719 | 387.185 | 4 |  | X | X | X | X |
| [KNR]  122-124 | [SBKIQK]  116-121 | K118+K122 | 1275.685 | 638.346 | 2 | X |  | X | X | X |
| [KNR]  122-124 | [SBKIQK]  116-121 | K118+K122 | 1275.684 | 425.900 | 3 | X |  | X | X | X |
| [KNR]  122-124 | [SBKIQK]  116-121 | K118+K122 | 1275.685 | 319.677 | 4 | X |  | X | X | X |
| [KNR]  122-124 | [SBKIQK]  116-121 | K118+K122 | 1279.710 | 638.346 | 2 |  | X | X | X | X |
| [KNR]  122-124 | [SBKIQK]  116-121 | K118+K122 | 1279.708 | 425.900 | 3 |  | X | X | X | X |
| [KNR]  122-124 | [SBKIQK]  116-121 | K118+K122 | 1279.708 | 319.677 | 4 |  | X | X | X | X |
| [KAR]  198-200 | [SILTGK]  201-206 | K198+S201 | 1087.647 | 544.327 | 2 | X |  | X |  | X |
| [KAR]  198-200 | [SILTGK]  201-206 | K198+S201 | 1087.647 | 363.220 | 3 | X |  | X | X | X |
| [KAR]  198-200 | [SILTGK]  201-206 | K198+S201 | 1091.672 | 546.340 | 2 |  | X | X | X | X |
| [KAR]  198-200 | [SILTGK]  201-206 | K198+S201 | 1091.671 | 364.562 | 3 |  | X | X |  | X |
| [KK]  197-198 | [SILTGK]  201-206 | K197/K198+S201 | 988.603 | 330.206 | 3 | X |  | X | X | X |
| [KK]  197-198 | [SILTGK]  201-206 | K197/K198+S201 | 992.628 | 331.548 | 3 |  | X | X | X | X |
| [NKBQYBR]  125-131 | {GAMEQPQEEAPEVR]  0-14 | {+K126 | 2694.167 | 898.727 | 3 | X |  | X |  |  |
| [NKBQYBR]  125-131 | {GAMEQPQEEAPEVR]  0-14 | {+K126 | 2698.195 | 900.070 | 3 |  | X | X |  |  |
| [nKBQYBR]  125-131 | {GAMEQPQEEAPEVR]  0-14 | {+K126 | 2699.175 | 900.396 | 3 |  | X | X |  | X |
| [MKLEYEK]  106-112 | {GAmEQPQEEAPEVR]  0-14 | {+K107 | 2626.234 | 876.083 | 3 |  | X | X |  | X |
| [MPEAEKR]  150-156 | {GAmEQPQEEAPEVR]  0-14 | {+K155 | 2546.179 | 849.398 | 3 |  | X | X |  | X |
| [MPEAEKR]  150-156 | {GAmEQPQEEAPEVR]  0-14 | {+K155 | 2542.158 | 848.058 | 3 | X |  | X |  |  |
| [IQKK]  119-122 | {GAMEQPQEEAPEVR]  0-14 | {+K121/K122 | 2182.083 | 728.032 | 3 | X |  | X | X | X |
| [IQKK]  119-122 | {GAMEQPQEEAPEVR]  0-14 | {+K121/K122 | 2186.107 | 729.374 | 3 |  | X | X |  |  |
| [IQKK]  119-122 | {GAmEQPQEEAPEVR]  0-14 | {+K121/K122 | 2198.079 | 733.365 | 3 | X |  | X |  | X |
| [KNR]  122-124 | {GAmEQPQEEAPEVR]  0-14 | {+K122 | 2098.987 | 700.334 | 3 | X |  | X | X | X |
| [KNR]  122-124 | {GAmEQPQEEAPEVR]  0-14 | {+K122 | 2103.007 | 701.674 | 3 |  | X | X |  | X |
| [KnR]  122-124 | {GAmEQPQEEAPEVR]  0-14 | {+K122 | 2099.968 | 700.661 | 3 | X |  | X |  | X |
| [KAR]  198-200 | {GAMEQPQEEAPEVR]  0-14 | {+K198 | 2039.984 | 680.666 | 3 | X |  | X |  |  |
| [KAR]  198-200 | {GAmEQPQEEAPEVR]  0-14 | {+K198 | 2055.978 | 685.997 | 3 | X |  | X |  | X |
| [KK]  197-198 | {GAmEQPQEEAPEVR]  0-14 | {+K197/K198 | 1956.929 | 652.981 | 3 | X |  | X | X | X |
| [KK]  197-198 | {GAMEqPQEEAPEVR]  0-14 | {+K197/K198 | 1941.923 | 647.979 | 3 | X |  | X | X | X |
| [IKK]  422-424 | [TETETSLHPLLQEIYKDmY}  425-444 | K423+K440 | 2810.420 | 937.478 | 3 | X |  | X |  | X |
| [KK]  197-198 | [MPEAEKR]  150-156 | K155+K197 | 1230.652 | 410.889 | 3 | X |  | X | X | X |
| [KK]  197-198 | [MPEAEKR]  150-156 | K155+K197 | 1234.676 | 309.424 | 4 |  | X | X |  | X |
| [KK]  197-198 | [MPEAEKRK]  150-157 | K155+K197/K198 | 1358.746 | 340.442 | 4 | X |  | X |  |  |
| [KK]  197-198 | [MPEAEKRK]  150-157 | K155+K197/K198 | 1362.770 | 341.448 | 4 |  | X | X |  |  |
| [KAR]  198-200 | [SBKIQK]  116-121 | K118+K198 | 1232.678 | 411.564 | 3 | X |  | X |  |  |
| [KAR]  198-200 | [SBKIQK]  116-121 | K118+K198 | 1236.702 | 412.906 | 3 |  | X | X |  |  |
| [mPEAEKR]  150-156 | [HIYNAYLKNFNMTK]  183-196 | K155+K190 | 2732.355 | 683.844 | 4 |  | X | X |  |  |
| [MPEAEKR]  150-156 | [HIYNAYLKNFNmTK]  183-196 | K155+K190 | 2732.342 | 683.841 | 4 |  | X | X |  |  |
| [MPEAEKR]  150-156 | [LLQKmADLR]  401-409 | K155+K404 | 2059.071 | 515.523 | 4 | X |  | X |  |  |
| [IQKK]  119-122 | [VBGDKASGFHYGVHABEGBK]  78-97 | K82+K121/K122 | 2854.340 | 952.118 | 3 |  | X | X |  |  |
| [KKAR]  197-200 | {GAMEQPQEEAPEVR]  0-14 | {+K197/K198 | 2168.081 | 723.365 | 3 | X |  | X |  | X |
| [KKAR]  197-200 | {GAMEQPQEEAPEVR]  0-14 | {+K197/K198 | 2172.102 | 724.706 | 3 |  | X | X |  |  |
| [KNR]  122-124 | [FQKBLALGMSHNAIR]  132-146 | K122+K134 | 2258.159 | 565.295 | 4 | X |  | X | X |  |
| [MPEAEKR]  150-156 | [MKLEYEKBER]  106-115 | K107+K155 | 2341.098 | 586.030 | 4 | X |  | X |  |  |
| [KNR]  122-124 | [KLVAGLTANEGSQYNPQVADLK]  157-178 | K122+K157 | 2828.494 | 707.879 | 4 | X |  | X |  |  |
| [KNR]  122-124 | [NKBQYBR]  125-131 | K122+K126 | 1544.737 | 515.583 | 3 |  | X |  | X |  |
| [KKAR]  197-200 | [SILTGK]  201-206 | K198+S201 | 1215.742 | 405.919 | 3 | X |  |  | X | X |
| [KNR]  122-124 | [FQKBLALGmSHNAIR]  132-146 | K122+K134 | 2274.161 | 569.296 | 4 | X |  |  |  | X |
| [KNR]  122-124 | [FQKBLALGmSHNAIR]  132-146 | K122+K134 | 2278.185 | 570.302 | 4 |  | X |  |  | X |
| [KK]  197-198 | [FQKBLALGmSHNAIR]  132-146 | K134+K197/K198 | 2136.135 | 534.789 | 4 |  | X |  |  | X |
| [KK]  197-198 | [MPEAEKR]  150-156 | K155+K197 | 1230.649 | 308.418 | 4 | X |  |  |  | X |
| [NKBQYBR]  125-131 | {GAmEQPQEEAPEVR]  0-14 | {+K126 | 2714.189 | 905.401 | 3 |  | X |  |  | X |
| [NKBQYBR]  125-131 | {GAmEQPQEEAPEVR]  0-14 | {+K126 | 2710.170 | 904.062 | 3 | X |  |  |  | X |
| [nKBQYBR]  125-131 | {GAMEQPQEEAPEVR]  0-14 | {+K126 | 2699.181 | 900.399 | 3 |  | X |  |  | X |
| [KnR]  122-124 | {GAmEQPQEEAPEVR]  0-14 | {+K122 | 2103.993 | 702.003 | 3 |  | X |  |  | X |
| [KNR]  122-124 | {GAMEQPQEEAPEVR]  0-14 | {+K122 | 2082.989 | 695.001 | 3 | X |  |  |  | X |
| [KnR]  122-124 | {GAMEQPQEEAPEVR]  0-14 | {+K122 | 2087.998 | 696.671 | 3 |  | X |  |  | X |
| [KK]  197-198 | {GAMEQPQEEAPEVR]  0-14 | {+K197/K198 | 1944.963 | 648.993 | 3 |  | X |  |  | X |
| [KK]  197-198 | {GAmEQPQEEAPEVR]  0-14 | {+K197/K198 | 1960.958 | 654.324 | 3 |  | X |  |  | X |
| [IKK]  422-424 | {GAMEQPQEEAPEVR]  0-14 | {+K423 | 2054.027 | 685.348 | 3 | X |  |  |  | X |
| [IKK]  422-424 | {GAmEQPQEEAPEVR]  0-14 | {+K423 | 2070.016 | 690.677 | 3 | X |  |  |  | X |
| [KKAR]  197-200 | [SILTGK]  201-206 | K198+S201 | 1219.767 | 407.261 | 3 |  | X |  |  | X |
| [KNR]  122-124 | [MPEAEKR]  150-156 | K122+K155 | 1372.699 | 343.930 | 4 | X |  |  |  | X |
| [SBKIQK]  116-121 | {GAmEQPqEEAPEVR]  0-14 | {+S116/K118 | 2450.153 | 817.389 | 3 |  | X |  |  | X |
| [KK]  197-198 | [FQKBLALGmSHNAIR]  132-146 | K134+K197/K198 | 2136.135 | 534.789 | 4 |  | X |  |  | X |
